# Improving the potency prediction for chemically modified siRNAs through insights from molecular modeling of individual sequence positions

**DOI:** 10.1016/j.omtn.2024.102415

**Published:** 2024-12-05

**Authors:** Evgenii Kliuchnikov, Farkhad Maksudov, Jeffrey Zuber, Sarah Hyde, Adam Castoreno, Scott Waldron, Mark K. Schlegel, Kenneth A. Marx, Martin A. Maier, Valeri Barsegov

**Affiliations:** 1 Department of Chemistry, University of Massachusetts, Lowell, MA 01854, USA; 2 Alnylam Pharmaceuticals, Cambridge, MA 02142, USA

**Keywords:** MT: Oligonucleotides: Therapies and Applications, RNA interference, siRNA, computational molecular modeling, statistical inference

## Abstract

Chemical modifications are applied to small interfering RNAs (siRNAs) to improve their metabolic stability, specificity, and duration of pharmacodynamic effects. Despite tremendous progress made, identifying chemically modified siRNAs with drug-like properties requires empirical screening due to an intricate interdependence of siRNA sequence and chemistry, i.e., the nature and position of chemical modifications within the siRNA duplex. To improve our ability to design fully modified, potent siRNAs, we combined experimental measurements of thermodynamic stability and biological activity *in vitro* with extensive molecular modeling *in silico* of the structural, dynamic, and energetic properties of parent (unmodified) siRNA duplex sequences compared with their chemically modified variants. A pattern of modifications at specific positions were identified, where the combination of sequence and chemical modifications play an outsized role in the observed biological activity. Molecular modeling revealed low stabilization energies and increased sugar stereochemical flexibility for 2′-F modified position g2 and less so for g6 in the guide strand seed region. Machine learning confirmed that these properties correlate with higher observed biological activity. These results provide molecular-level insights into the effects of chemical modifications on the intrinsic activity of siRNAs, which can be used in the rational design of chemically modified siRNAs with uncompromised potency.

## Graphical abstract

**Figure:**
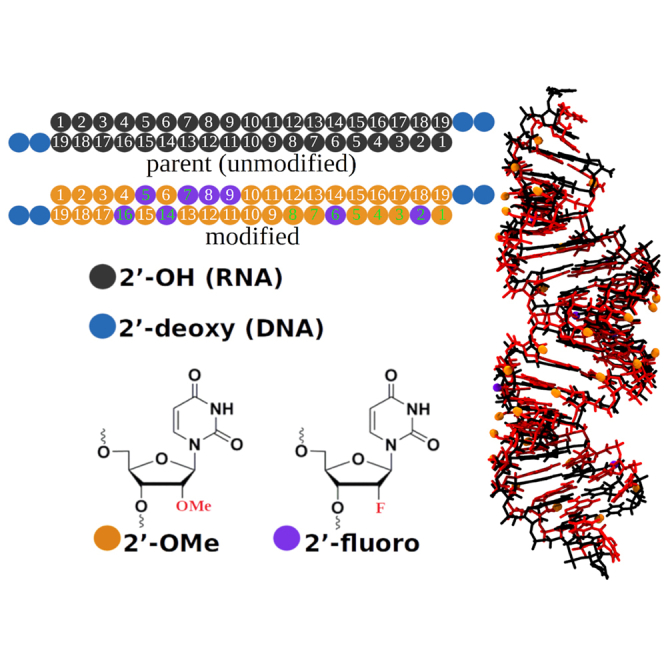

## Teaser

Barsegov, Maier, and colleagues combined *in vitro* experiments of thermodynamic stability and biological activity with *in silico* modeling of the structural, dynamic, and energetic properties of siRNA duplexes to show the effects of chemical modifications on siRNA activity. The results obtained allow design of chemically modified siRNAs with uncompromised potency.

## Introduction

RNA interference (RNAi) is a complex multistep process that post-transcriptionally controls gene expression in cells.^1^ A small interfering RNA (siRNA) duplex is loaded into the precursor RNA-induced silencing complex (pre-RISC) a large multi-component assembly containing argonaute 2 (Ago2). Upon RISC activation, the siRNA passenger strand is removed, while the guide strand remains bound within the Ago2 protein.^2^ The siRNA-guided RISC has the ability to bind to a complementary site on the target messenger RNA (mRNA) and suppress its translation via endonucleolytic cleavage,^3^ which results in subsequent degradation of the mRNA.^4^ Since the discovery of RNAi in 1998,^1^ it has become evident that this naturally occurring mechanism of RNAi holds immense therapeutic potential to suppress the expression of disease-associated genes via post-transcriptional silencing. Consequently, RNAi therapeutics have emerged as a new class of medicines that have already been utilized to address several unmet medical needs. The first ever RNAi drug was approved by the Food and Drug Administration in 2018^5^^,^^6^ and since then several other drugs have obtained regulatory approval for the treatment of a wide variety of diseases.^7^^,^^8^^,^^9^

Unmodified siRNAs have several limitations, including metabolic instability, a potential for innate immune activation, and inefficient cellular uptake, which prohibit their use as drugs. To improve the drug-like properties of siRNAs, chemical modifications primarily focused on the sugar-phosphate backbone have been introduced to enhance nuclease resistance, improve RNAi specificity, and optimize the duration of effect.^10^^,^^11^^,^^12^^,^^13^ Of the hundreds of different nucleic acid modifications explored in the context of siRNAs, only a handful have transitioned into development, including 2′-*O*-methyl, 2′-fluoro,^14^ phosphorothioate,^15^ glycol nucleic acid (GNA),^16^ and 5′-(*E*)-vinylphosphonate (VP).^17^^,^^18^ However, even after decades of research and the development of potent, relatively generalizable designs, it is not well understood how different chemical substitutions at each of the positions in the passenger and guide strands affect the intrinsic activity, i.e., ability of the siRNA strands to engage with RISC during the loading process and subsequent cleavage of its target mRNA. Specifically, the factors governing the interplay between sequence and chemistry of the siRNA and the resulting sequence-dependent tolerance for chemical modifications, remain poorly understood.

Advancements in high-performance computing,^19^^,^^20^^,^^21^^,^^22^ in molecular dynamics (MD) simulations applied to large biomolecules^23^^,^^24^ and in force field developments for RNA,^25^^,^^26^^,^^27^^,^^28^^,^^29^ have enabled theoretical exploration of siRNA duplexes on long, biologically relevant, microsecond timescales.^30^^,^^31^^,^^32^ Published reports have highlighted the use of computational molecular modeling approaches in designing and describing chemically modified siRNAs.^33^^,^^34^^,^^35^^,^^36^^,^^37^^,^^38^^,^^39^ For example, the effect of the 5′-end in the RNA guide strand has been investigated.^34^ The authors showed how different modifications at the 5′-end affect the binding affinity of the guide strand with the MID domain of hAgo2. The effect of 3′-overhangs in the guide strand has also been studied, focusing on the binding affinities between different modified 3′-overhangs and the PIWI/Argonaute/Zwille (PAZ) domain of Ago2.^35^ Modifications in the seed region were addressed in a study that showed no significant change in the stability of the Ago-RNA complex.^36^ The all-atom MD simulations have also been employed to analyze the structures and energies of siRNA, Ago2, and siRNA-Ago2 complex. In a recent study, Harikrishna et al. performed structural and thermodynamic analysis for Ago2 protein without RNA, Ago2 with guide strand loaded, and Ago2 with siRNA in the duplex form.^38^ In another recent study, RNA oligonucleotides containing a 7-membered sugar ring (oxepane nucleotides) were explored.^40^ In this study, the MD simulations were used to probe base stacking and sugar-phosphate hydrogen bonds.

While computational molecular modeling plays an important role in our understanding of biomolecules (DNA, RNA, and proteins), few MD simulation studies reported to date investigate the role of chemical modifications in the siRNA backbone on the conformational dynamics and thermodynamic stability of the parent vs. chemically modified siRNA duplexes,^41^ and no studies to date have taken a position-specific view of backbone chemical changes and their effects on activity. To address some of these limitations and advance the rational design of chemically modified siRNA, we have combined experimental studies of duplex stability and siRNA activity with the all-atomic computational molecular modeling. We carried out computational modeling tasks on pairs of parent (unmodified) and chemically modified siRNAs to explore the interplay among their structure, dynamics, energetics, and biological activity. For this study, we focused on a well-established fully 2′-*O*-methyl/2′-fluoro chemistry pattern, which has been optimized for potency and duration in the context of siRNA conjugates.^13^ To avoid complicating the analysis with additional design features, we selected a “standard” 21/21 mer design with universal dTdT overhangs on both strands. A total of 15 pairs of siRNA duplexes were selected, 11 pairs targeting the *SERPINA6* transcript and four pairs targeting the *AGT* transcript. Each pair was composed of the parent unmodified siRNA duplex matched with its chemically modified counterpart. This set of compounds was selected from a larger initial pool to include examples exhibiting a substantial potency loss for the modified siRNA compared with their unmodified counterparts as well as pairs with excellent potency translation.

Using the output from the MD simulations, we were able to describe the structures, conformational dynamics, thermodynamic properties, and sugar stereochemistry of native siRNA duplexes, as well as their position-dependent properties, compared with their chemically modified ribose counterparts. The results reveal molecular-level insights into the effect of sequence in the context of a specific chemical modification pattern on the intrinsic activity of siRNAs and provide interpretation of the experimental data, which includes thermal duplex stability ($T_{m}$) and siRNA activity measurements *in vitro*. Theoretically estimated guide-passenger strand interaction energies correlate very well with predictions of the empirical nearest-neighbor model, as well as with measured half maximal inhibitory concentration (IC_50_) and $T_{m}$ data. Statistical analysis of the parameters derived from a large set of 1-μs long MD simulations allowed us to identify a feature subset, that is predictive of potency translation, and to illuminate the importance of guide strand positions, in particular position 2 in the seed region harboring a C2′-F sugar with low negative interaction energy leading to enhanced stereochemical flexibility. These properties were found to be correlated with increased biological activity in the modified sequences. These results help to explain why certain siRNAs lose activity while other siRNAs do not, when a well-established chemical modification pattern is applied, and shed light on the inter-dependency of siRNA sequence and chemistry to guide sequence-specific chemistry optimization.

## Results

### IC_50_ data for parent and modified siRNA duplexes

**Figure 1:**
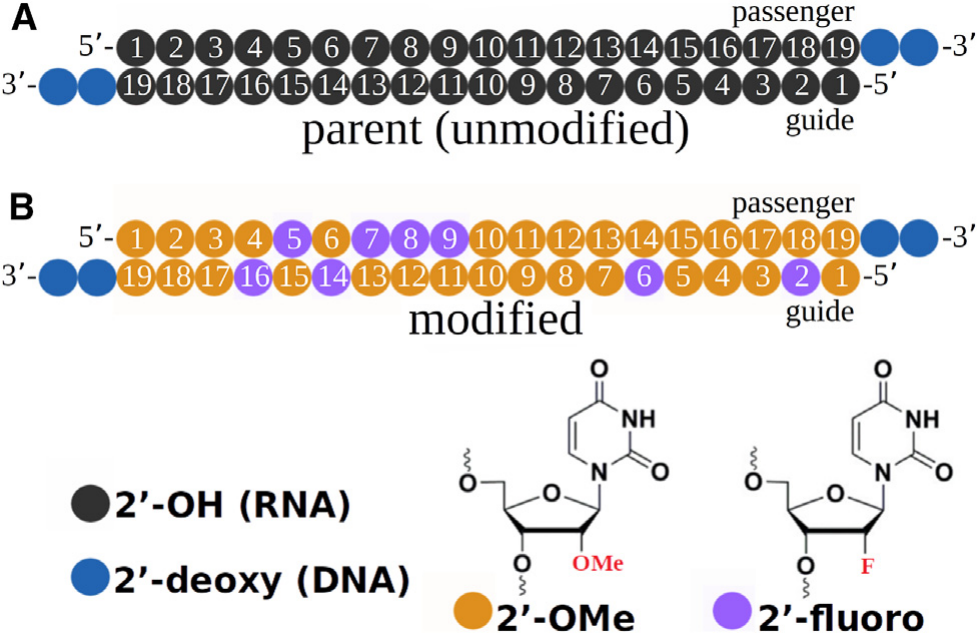
siRNA structure and chemistries (A) Structure of canonical (2′-OH) nucleobases in parent (unmodified) siRNA sequences. (B) siRNA sequences with the 2′-sugar modifications 2′-*O*-methyl (2′-OMe) and 2′-fluorine (2′-F) in the guide and passenger strand positions: 2′-OMe (2′-F) is shown in orange (purple) color. DNA base overhangs at the 3′-end in the guide and passenger strands are depicted in blue color. All siRNA sequences are listed in Table 1.

The IC_50_ values for 15 siRNA pairs (unmodified parent and chemically modified; see Figure 1), four targeting the human transcript of *AGT* and 11 targeting *SERPINA6*, were measured *in vitro* to determine the impact of a fully modified chemical modification pattern on siRNA potency (Table 1). The siRNAs were selected from a larger initial pool to cover a wide range of potencies. Six sequences showed a substantial loss of activity with the application of the chemistry template (IC_50_ >100 nM); four displayed a moderate loss of activity (>10x but with measurable IC_50_) and the remaining five sequences showed no impact or even a slight gain in potency with the chemical modification pattern applied. The range of responses to chemical modifications made this set of siRNA pairs suitable for a computational interrogation of duplex structural properties, conformational dynamics, and thermodynamic characteristics. In the following analyses, we separated the modified sequences into “active” and “inactive” based on the following criteria: the IC_50_ ratio (modified/parent) for “active” siRNAs is less than 100 with a measurable IC_50_ while “inactive” siRNAs exhibit a greater 100x increase in IC_50_ and/or an IC_50_ value of greater than 100 nM (not measurable).

**Table 1:** siRNA sequences with experimental IC_50_ and $T_{m}$ data

siRNA ID	Passenger	Guide	IC_50_, nM	$T_{m}$,°C
siSER-1	ACCAGCGGCCUCUGGACCAdTdT	UGGUCCAGAGGCCGCUGGUdTdT	4.4	75.5
siSER-1m	accaGfcGfGfCfcucuggaccadTdT	uGfgucCfagaggccGfcUfggudTdT	>100	86.7
siSER-2	CUCCCCUGUGAGCAUCUCAdTdT	UGAGAUGCUCACAGGGGAGdTdT	0.11	68.5
siSER-2m	cuccCfcUfGfUfgagcaucucadTdT	uGfagaUfgcucacaGfgGfgagdTdT	27.2	78.5
siSER-3	CCCAGCUUCUCCAGGGCCUdTdT	AGGCCCUGGAGAAGCUGGGdTdT	0.33	74
siSER-3m	cccaGfcUfUfCfuccagggccudTdT	aGfgccCfuggagaaGfcUfgggdTdT	>100	86.1
siSER-4	UUGCUGGAGUCAUUCUCAAdTdT	UUGAGAAUGACUCCAGCAAdTdT	0.032	59.5
siSER-4m	uugcUfgGfAfGfucauucucaadTdT	uUfgagAfaugacucCfaGfcaadTdT	0.027	69.7
siSER-5	AGACAUCAAGCACUACUAUdTdT	AUAGUAGUGCUUGAUGUCUdTdT	0.2	56
siSER-5m	agacAfuCfAfAfgcacuacuaudTdT	aUfaguAfgugcuugAfuGfucudTdT	0.23	66.3
siSER-6	UCCCCUGCCAGCUGGUGCAdTdT	UGCACCAGCUGGCAGGGGAdTdT	2.74	76
siSER-6m	ucccCfuGfCfCfagcuggugcadTdT	uGfcacCfagcuggcAfgGfggadTdT	>100	87.7
siSER-7	AGGUCACCAUCUCUGGAGUdTdT	ACUCCAGAGAUGGUGACCUdTdT	0.56	65.1
siSER-7m	agguCfaCfCfAfucucuggagudTdT	aCfuccAfgagauggUfgAfccudTdT	>100	78.1
siSER-8	UCACCUGGAGCAGCCUUUUdTdT	AAAAGGCUGCUCCAGGUGAdTdT	1.3	67.1
siSER-8m	ucacCfuGfGfAfgcagccuuuudTdT	aAfaagGfcugcuccAfgGfugadTdT	0.15	77.6
siSER-9	CUGACUUUGGGAACCAGGAdTdT	UCCUGGUUCCCAAAGUCAGdTdT	0.16	63.2
siSER-9m	cugaCfuUfUfGfggaaccaggadTdT	uCfcugGfuucccaaAfgUfcagdTdT	>100	73.7
siSER-10	AAGUUCUUCUCCCUCCAAAdTdT	UUUGGAGGGAGAAGAACUUdTdT	0.001	61.3
siSER-10m	aaguUfcUfUfCfucccuccaaadTdT	uUfuggAfgggagaaGfaAfcuudTdT	0.004	71.3
siSER-11	ACUUUAGGCAUCUUUUAAUdTdT	AUUAAAAGAUGCCUAAAGUdTdT	0.0007	46.3
siSER-11m	acuuUfaGfGfCfaucuuuuaaudTdT	aUfuaaAfagaugccUfaAfagudTdT	0.0001	56.1
siAGT-1	CCUGGCUGCAGGUGACCGAdTdT	UCGGUCACCUGCAGCCAGGdTdT	0.04	72.9
siAGT-1m	ccugGfcUfGfCfaggugaccgadTdT	uCfgguCfaccugcaGfcCfaggdTdT	>100	84.5
siAGT-2	AGCAAUGACCGCAUCAGGAdTdT	UCCUGAUGCGGUCAUUGCUdTdT	0.13	64
siAGT-2m	agcaAfuGfAfCfcgcaucaggadTdT	uCfcugAfugcggucAfuUfgcudTdT	4.9	74.1
siAGT-3	CAAAAAUUGGGUUUUAAAAdTdT	UUUUAAAACCCAAUUUUUGdTdT	0.0004	39
siAGT-3m	caaaAfaUfUfGfgguuuuaaaadTdT	uUfuuaAfaacccaaUfuUfuugdTdT	0.022	47.2
siAGT-4	GGGUGGGGAGGCAAGAACAdTdT	UGUUCUUGCCUCCCCACCCdTdT	0.01	73.5
siAGT-4m	ggguGfgGfGfAfggcaagaacadTdT	uGfuucUfugccuccCfcAfcccdTdT	0.21	83.8

siRNA sequence and chemical modifications are displayed as follows: RNA: upper case, 2′-OMe: lower case; and 2′-F: Nf (‘N’ being either A, C, G, or U). The $T_{m}$ and IC_50_ values were determined experimentally (see materials and methods). The corresponding target transcripts *SERPINA6* and angiotensinogen are indicated in the siRNA IDs with *SERPINA6* and *AGT* targets, respectively.

### Melting temperatures for parent and modified siRNA duplexes

To study the impact of the chemical modifications on duplex stability, we measured the melting temperatures $\left(T_{m}\right)$, i.e., the temperature at which 50% of the duplexes dissociate into the guide and passenger strands, for this set of parent and modified siRNAs (Table 1). For every siRNA pair, the chemical modifications increased $T_{m}$ by an average of 10°C, ranging between 7°C and 12°C.

### Quantum chemistry calculations and force field development for modified siRNA

To correlate the experimentally determined biological activity and thermodynamic properties with the structural and physicochemical properties of the siRNA duplexes at the molecular level, we turned to computational molecular modeling. Although force fields for unmodified nucleic acids (DNA and RNA) are available, we had to develop the force fields for describing double-stranded RNAs (dsRNAs) with 2′-F and 2′-OMe ribose modifications (see supplemental information). Quantum chemistry calculations for the sugar pucker (C2′-endo and C3′-endo conformations) yielded a 0.7–1.4 kcal/mol free-energy difference between these conformations, which agrees with the literature.^42^ Hence, in the calculation of atomic partial changes, construction of the force field parameters, and in the all-atom MD simulations, we set all ribonucleotides to be in the free-energy minimum C3′-endo conformation (Figure S1). *Atomic partial charges* were calculated for atoms in a chemically modified ribose ring in the C3′-endo conformation (supplemental methods). The atomic partial charges are displayed in Figures S1A and S1B. *Force field parameters* were derived as described in materials and methods. All molecular mechanics parameters are accumulated in Tables S1 and S2.

### Secondary and tertiary structures of parent vs. chemically modified siRNA duplexes

First, we probed the effect of chemical modifications on the secondary structures of siRNA duplexes compared with those of the corresponding unmodified siRNAs (Figure 1; Table 1). For most siRNA sequences evaluated here, the base pairing and base stacking parameters at most positions do not change significantly (see supplemental information) when comparing parent and modified duplex structures (see example for siSER-10 in Figure S2).

**Figure 2:**
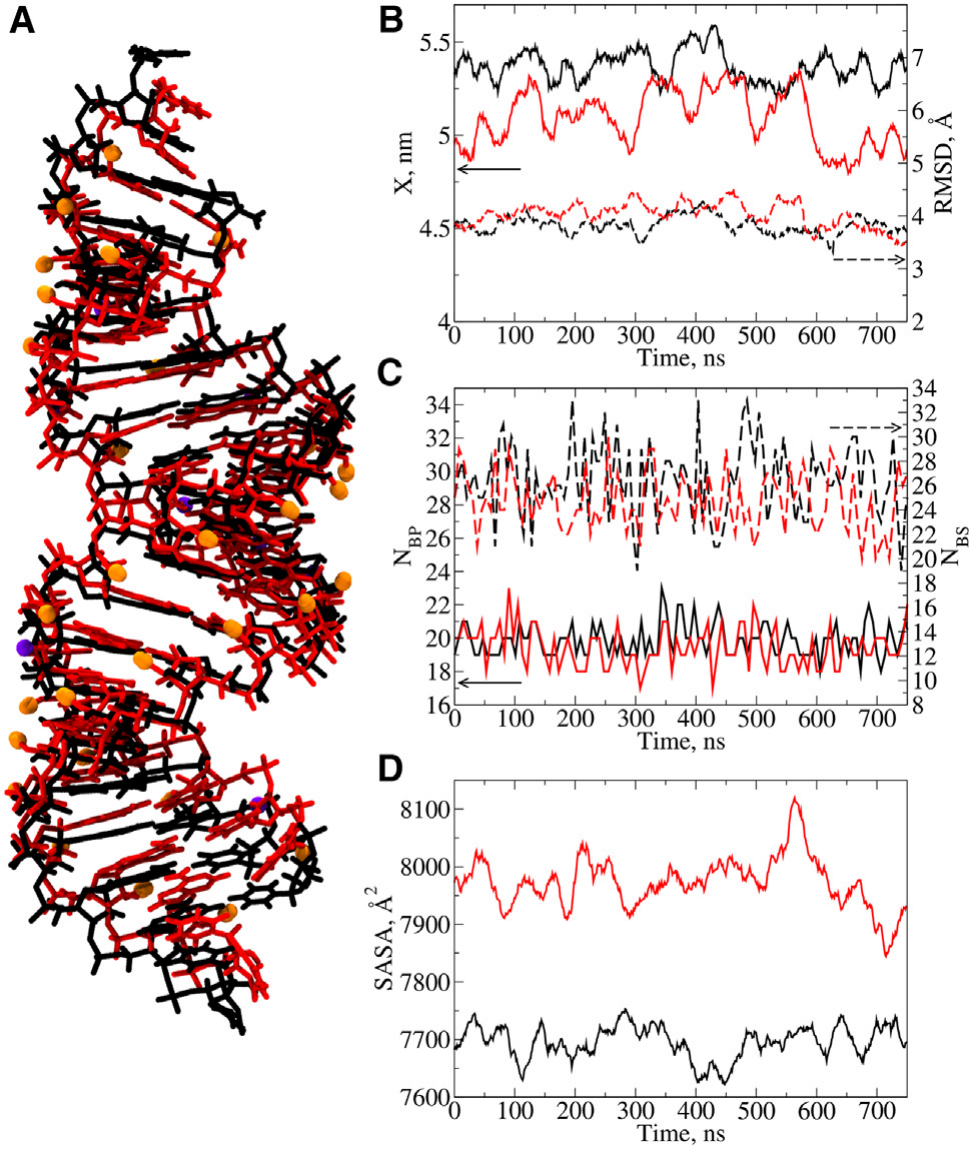
Dynamic molecular properties of siRNA duplexes siSER-10 and siSER-10m (A) Average atomic structures of parent siSER-10 (black) and modified dsRNA siSER-10m (red) with 2′-ribo substitutions shown by orange (2′-OMe) and purple (2′-F) spheres. (B) Time profiles of the end-to-end distance X (solid lines, left y axis) and root-mean-square deviation RMSD (dashed lines, right y axis) for siRNA duplexes siSER-10 (black) and siSER-10m (red). (C) Number of base pairs $N_{BP}$ (solid lines, left y axis) and number of base stacks $N_{BS}$ (dashed lines, right y axis) in siRNA duplexes siSER-10 (black) and siSER-10m (red) vs. time. (D) Time profile of solvent accessible surface area (SASA) for siRNA duplexes siSER-10 (black) and siSER-10m (red).

Next, we probed the tertiary structures of siRNA duplexes. Representative time profiles of the end-to-end distance X, the root-mean-square deviations (RMSD), the total number of base pairs $N_{BP}$ and base stacks $N_{BS}$ reinforcing the duplex arrangement, and the solvent accessible surface area (SASA) calculated for parent and modified siSER-10 are displayed in Figure 2. The overall trend for all siRNAs is that the parent structures are ∼0.1–0.2 nm longer than their modified counterparts (Figure 2B) with only a few exceptions (Table S3). Modified siRNAs exhibit lower RMSD values than their parent siRNA counterparts (Figure 2B), with siSER-10 being a notable exception. The numbers of base pairs $N_{BP}$ and base stacks $N_{BS}$ are comparable for all siRNA pairs with $N_{BP}$ fluctuating between 18 and 23, and $N_{BS}$ between 20 and 32 (Figure 2C). There are $N_{BP}=$ 19 bases from each strand that are expected to form Watson-Crick base pairing in any of the siRNAs analyzed in this work; however, $N_{BP}$ is higher than 19, because the overhangs at the 3′- and 5′-end are also capable of forming hydrogen-bonded base pairs with their nearest-neighbor nucleobases. For parent siSER-10, SASA (describing the degree to which a molecule is exposed to water) averages 7,529 Å^2^ while siSER-10m averages 7,950 Å^2^ (Figure 2D). For all siRNAs, the introduction of chemical modifications increases the average SASA by ∼300-400 Å^2^ (Table S3), because the modified siRNAs form wider structures compared with the parent sequences. To better understand the origin of the increased SASA for modified siRNAs, we analyzed several parameters. The average molecular volume V of modified siRNAs is greater by about 200 Å^3^ and the average density ρ of modified siRNAs is about 0.07 g/mL greater than their corresponding parent sequences. Taken together, these results indicate that modified duplex siRNAs are shorter but wider, have greater SASA and volume, and are denser than their corresponding parent sequences due to the added mass of the modified sugars.

### Thermodynamic stability of parent vs. chemically modified siRNA duplexes

We estimated the thermodynamic stability of siRNA duplexes through the energy changes associated with formation of each structure from their guide and passenger strands. The values of electrostatic energy of interaction (Coulombic coupling between charged atoms) $E_{el}$, the van der Waals energy (excluded volume interactions) $\Delta E_{vdW}$, and solvation energy (polar solvation of charged atoms plus hydrophobic interactions) ${\Delta E}_{solv}$ for all siRNAs are listed in Table S4. As expected, $\Delta E_{solv}$ is a major driving force for duplex formation, and it compensates the unfavorable interactions between charged phosphate groups ($\Delta E_{el})$. The inclusion of chemical modifications increases $\Delta E_{solv}$, implying better solubility. The excluded volume interactions are more favorable for modified compared with parent siRNAs with $\Delta E_{vdW}$ increases of −17 kcal/mol (on average) for modified siRNA duplexes, which correlate well with their increase in RNA mass densities ρ (Table S3), implying denser packing. Analysis of the changes in energy ΔE and entropy TΔS for formation of the siRNA duplex structure indicate that chemically modified siRNA duplexes display stronger guide-passenger strand coupling (Table S4). The presence of the 2′-modifications decreases ΔE by 9 kcal/mol on average (stronger interaction), lowers TΔS by 4 kcal/mol on average (more ordered structures) and decreases ΔG by an average of 14 kcal/mol (increased stability) compared with parent siRNAs.

### Comparing theoretical calculations of thermodynamics with predictions of nearest-neighbor model

The empirical nearest-neighbor (NN) model^43^ is widely used to estimate the thermodynamic quantities associated with formation of parent RNA duplexes. Here, we used the NN model (see supplemental methods) to assess the accuracy of theoretical calculations of the interaction energy (enthalpy) ΔE for siRNAs (Table S4). Figure S3 compares the results of MD-based calculation of ΔE with the NN-model-based predictions of ΔE for both parent and modified siRNAs. The theoretically calculated quantities correlate very well with the NN-model-based predictions of ΔE particularly for the unmodified parent sequences, since the NN model is parameterized using measured energetics of sequences composed of only unmodified ribonucleotides (Figure S3A). The theoretically calculated ΔE also correlates well with the measured ln[IC_50_] (R= −0.8289) and $T_{m}$ (R= −0.9261) (data from Tables 1 and S4).

### Binary correlations between experimental data and theoretical quantities from MD simulations

**Figure 3:**
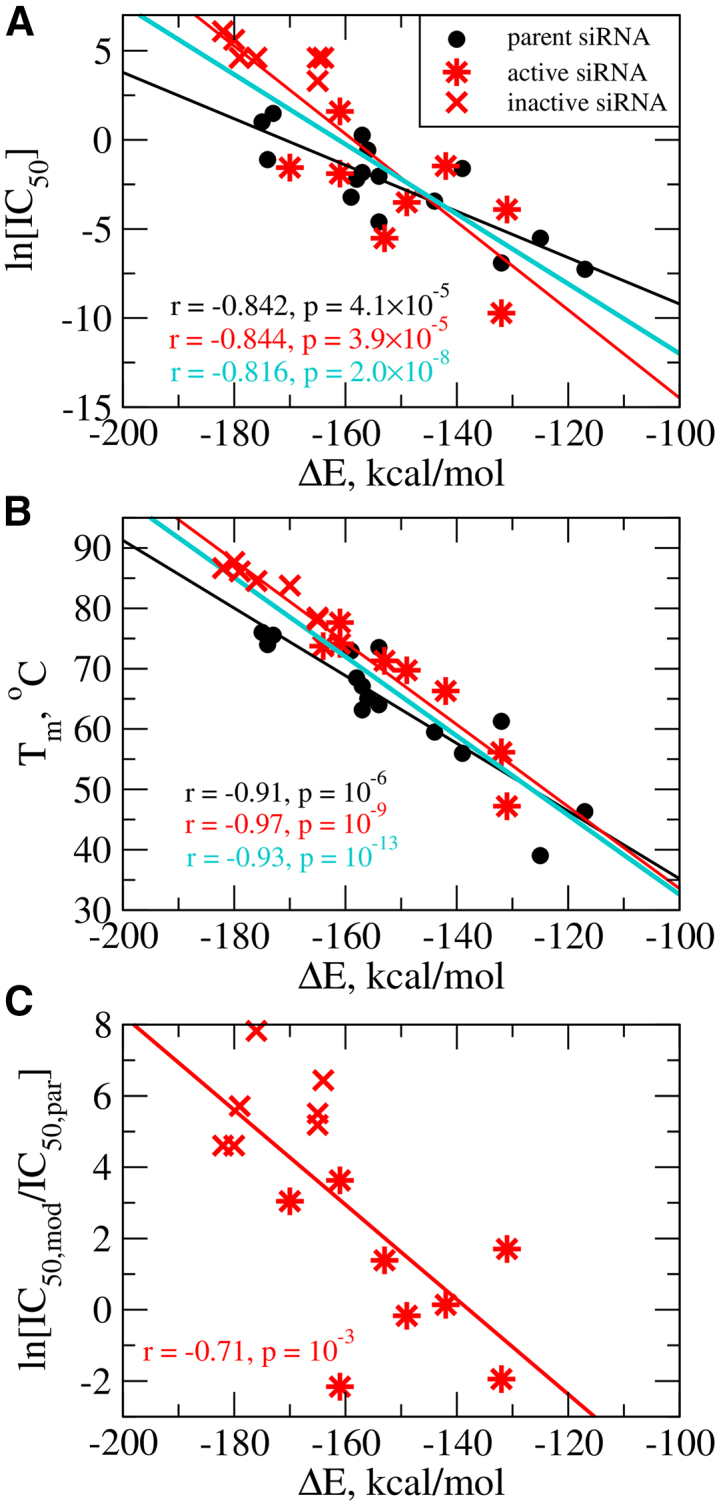
Correlations between $T_{m}$ and IC_50_ data and calculated values of interaction energy ΔE Profiles of the logarithm of IC_50_ vs. ΔE (A) and $T_{m}$ vs. ΔE (B) for parent (black), modified (red), and all (parent and modified, cyan) 15 siRNAs. Black circles represent parent siRNAs; star symbols represent the modified siRNAs that after their chemical substitution remain active; and crosses represent the modified siRNAs that after their substitution become inactive. Shown are values of the Pearson correlation coefficient r and the p-values calculated for parent siRNAs (black), for modified siRNAs (red), and for parent and modified siRNAs combined (cyan). Panel C: Profile and scatterplot of the logarithm of the IC_50_ ratio for modified relative to parent (unmodified) siRNA ln[IC_50,mod_/IC_50,par_] vs. ΔE. Also shown are values of the Pearson correlation coefficient r and the p-values (red).

Next, we addressed the question of whether siRNA parameters accessible through the MD simulations (Tables S3 and S4) correlate with experimentally measurable quantities (ln[IC_50_], $T_{m}$). The maps of binary correlations between various characteristics for all 15 siRNA pairs indicate that both positive and negative correlations exist (Figure S4). Strong correlations between the experimental siRNA potency ln[IC_50_] and calculated duplex ΔE values, and between $T_{m}$ and ΔE values are observed (Figures 3A and 3B). The inactive siRNAs cluster around higher $T_{m}$ and ΔE values. To investigate whether the relative changes in siRNA potency due to chemical modifications correlate to the duplex thermodynamics, we plotted the relative activity ln[IC_50,mod_/IC_50,par_] as a function of ΔE for the modified siRNAs (Figure 3C). A higher duplex stability tends to increase the potency loss for the modified siRNAs.

### Interaction energy ΔE decomposition and SASA on a per base pair basis

**Figure 4:**
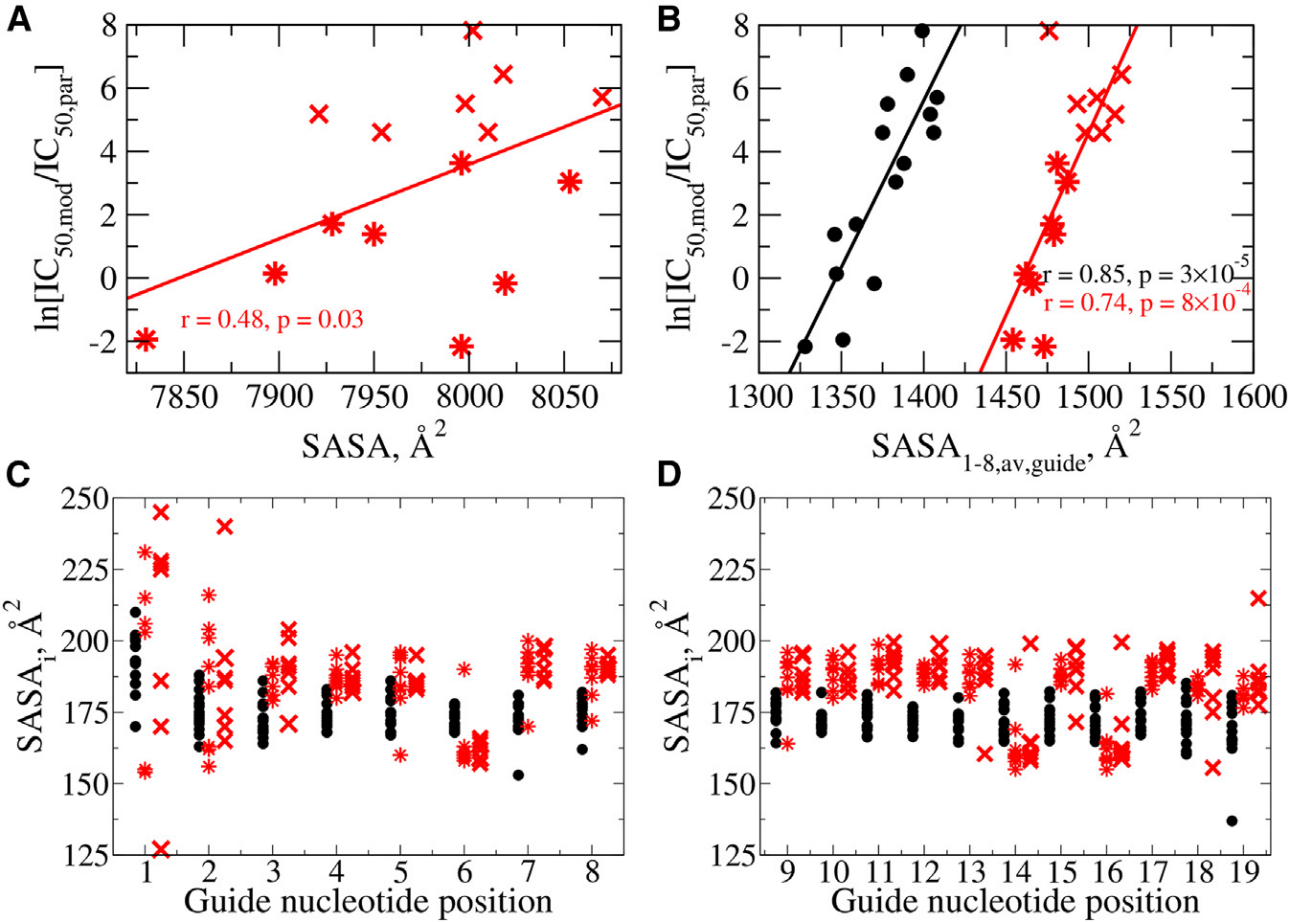
Correlations between SASA and relative potencies and SASA as function of nucleotide position (A) Relationship of IC_50_ ratio for modified relative to parent siRNA ln[IC_50,mod_/IC_50,par_] vs. full duplex SASA values. (B) Scatterplot and profiles of ln[IC_50,mod_/IC_50,par_] vs. the average SASA value for the guide strand positions g1–g8 ${SASA}_{1-8,av,guide}$ for parent (black circles) and modified (red stars for active red crosses inactive) siRNAs. Also shown are values of the Pearson correlation coefficient r and the p-values calculated for parent (black) and modified siRNAs (red). (C and D) Profiles of ${SASA}_{i}$ vs. nucleotide guide strand position g1–g8 in the seed region (C), and ${SASA}_{i}$ vs. nucleotide guide strand positions g9–g19 (D). Black circles represent the parent siRNAs, star symbols represent the modified active siRNAs, and crosses represent the modified inactive siRNAs.

The relationship between SASA values and relative potencies for the modified siRNAs is depicted in Figure 4A. The moderate correlation observed for ln[IC_50,mod_/IC_50,par_] and SASA led us to perform a more detailed, position-dependent analysis of the individual nucleotide positions i= g1-g19. The results for positions g1–g8 show that modified siRNA duplexes are reinforced by stronger interactions between the guide and passenger strands (ΔE) and higher SASA values compared with their parent unmodified siRNAs (Tables S5 and S6). To explore the origin of these effects in more detail, we analyzed the position-dependent interaction energy values $E_{i}$ for all 15 modified siRNAs (Figure S5). The $\Delta E_{i}$ values are consistently lower magnitude (less negative) quantities across all positions (lower stability) for the active siRNAs compared with the ones exhibiting a significant loss of activity following chemical modification. However, the differences in $\Delta E_{i}$ approach statistical significance only at positions g2 and g6 and, possibly, g15 and g17.

Similarly, we calculated the average SASA value for the guide strand positions g1–g8 (${SASA}_{1-8,av,guide}$) and plotted the relative activity of ln[IC_50,mod_/IC_50,par_] as a function of ${SASA}_{1-8,av,guide}$ for both parent and modified siRNAs (Figure 4B). The well-separated curves for parent and modified siRNAs illustrate the significant SASA increases for the modified siRNAs. In line with the previous observation for the average total SASA values, the more active siRNAs trend toward a lower average ${SASA}_{1-8,av,guide}$ compared with the less active ones. Training a statistical model, described in the supplemental information, to predict ln[IC_50_] from computed features of the duplexes also highlighted the various SASA measurements (SASA, ${SASA}_{1-8,av,guide+passenger}$, and ${SASA}_{1-8,av,guide}$) as predictive of ln[IC_50_], with all three featuring in the top 20 features and SASA identified as the top feature (see Figure S6). These clear differences between parent and modified sugar guide strand SASA values and the correlation with potency changes led us to perform a more detailed positional analysis of SASA across the entire guide strand (Figures 4C and 4D). Except for positions g1 and g2, the ${SASA}_{i}$ values for the modified guide strand siRNAs are generally clustered higher than for their parent counterparts for all 2′-OMe positions and lower for all 2′-F positions (g6, g14, and g16). Despite large positional SASA differences, we found that SASA values at individual positions (${SASA}_{i}$) could not be used to predict relative potency.

### Positional effects of base type and $\Delta E_{i}$ levels on siRNA activity

**Figure 5:**
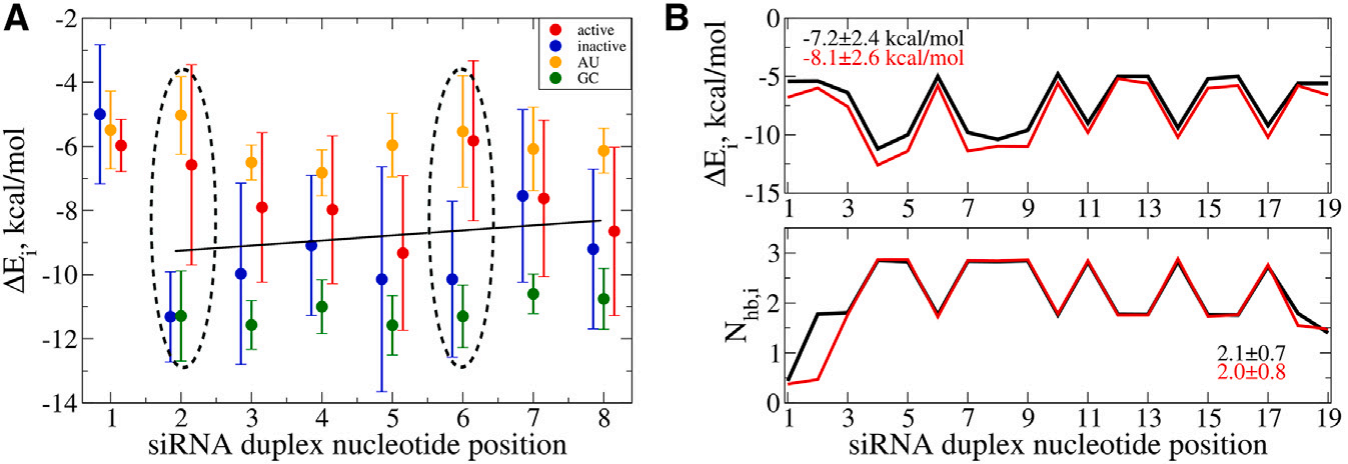
Base pair and position-dependent profiles of molecular properties (A) Interaction energy per base pair $\Delta E_{i}$ (averages and standard deviations) for the seed region (positions i= g1–g8), for active (red circles) and inactive (blue circles) modified siRNA sequences. Also shown are the averages and standard deviations for all the G and C bases combined (green circles), and for all the A and U bases combined (orange circles) at each position. Black line shows the linear SVM separation between active and inactive siRNAs. Positions with the largest separation between active and inactive are encircled. (B) Profiles of the average interaction energy $\Delta E_{i}$ per base pair (upper subpanel) and number of hydrogen bonds $N_{hb,i}$ per base pair (lower subpanel) for the nucleotides i= g1–g19 for parent siSER-10 (black curves) and modified siSER-10m (red curves). The average values and standard deviations for $\Delta E_{i}$ and $N_{hb,i}$ are shown in the graphs.

The observed position-dependent effects of ΔE and SASA on the activity levels of modified siRNAs suggested that the sequence/base composition could play a significant role. Here, we explore in greater detail the role of nucleobase type and their calculated $\Delta E_{i}$ values at different positions on activity across the seed region guide strand positions i= g1–g8 (Figure 5A), as well as all guide strand positions i= g1–g19 (Figure S5). The results indicate that position-dependent differences exist between active and inactive siRNAs with g2 and g6 positions showing the largest separations in average $\Delta E_{i}$ values, with less significant differences observed for positions g15 and g17 and minor differences for all other positions. Since in all subsequent analyses we observed only interesting differences for seed region positions g2 and g6, in the results and discussion sections that follow, we focus our attention only on these positions. While the Figure S5 positional differences are not evident in the data for the siSER-10 pair, a representative example of an “active” sequence (Figure 5B, upper panel), both parent and modified sequences have the lower average magnitude negative $\Delta E_{i}$ values characteristic of an active modified siRNA. The only notable difference in the number of H-bonds between parent and modified siRNA was observed at position g2 (Figure 5B, lower panel), implying an increased flexibility at this position for the modified siSER-10m compared with the parent sequence.

**Figure 6:**
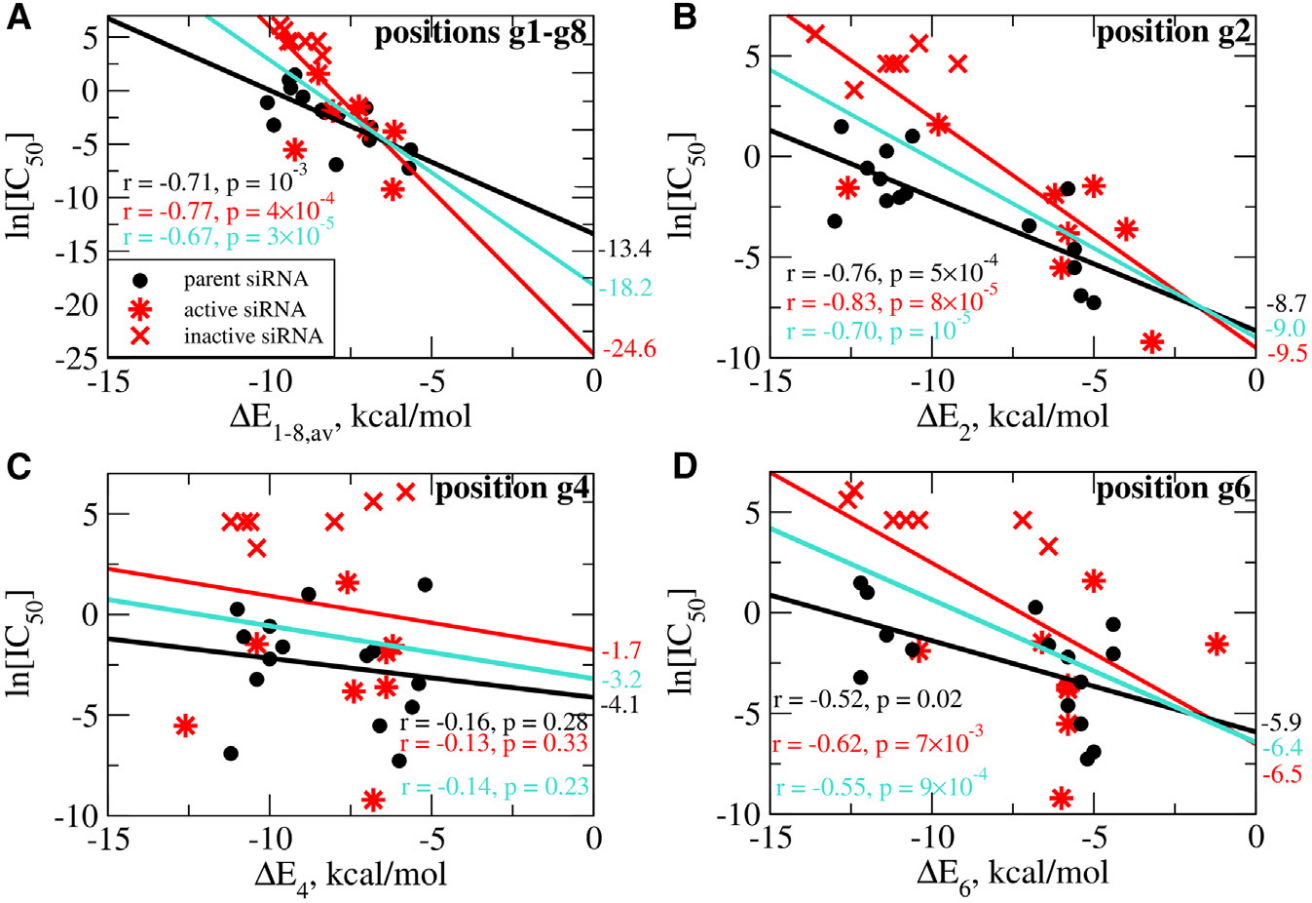
Correlations between experimental IC_50_ data and calculated thermodynamic molecular properties in the seed region Profiles of the logarithm of IC_50_ vs. average interaction energy for the seed region ${\Delta E}_{1-8,av}$ (A), vs. interaction energy for position g2 ${\Delta E}_{2}$ (B), vs. interaction energy for position g4 ${\Delta E}_{4}$ (C), and vs. interaction energy for position g6 ${\Delta E}_{6}$ (D) for parent (black), modified (red), and all (parent and modified, cyan) siRNAs. Black circles represent parent siRNAs, star symbols represent the modified active siRNAs, and crosses represent the modified inactive siRNAs. Also shown are values of the Pearson correlation coefficient r and the p-values calculated for parent siRNAs (black), for modified siRNAs (red), and for parent and modified siRNAs combined (cyan). The values on the right axes represent intercept values of the linear fits of the data for logarithm of IC_50_ at ${\Delta E}_{i}=$ 0 for parent siRNAs (black), for modified siRNAs (red), and for parent and modified siRNAs combined (cyan).

The position-specific energy decomposition (Figure 5A) suggests that certain positions have greater impact on activity than others. Although ln[IC_50_] values are correlated with the values of interaction energy for the seed region ${\Delta E}_{1-8,av}$ (Figure 6A), the position-specific $\Delta E_{i}$ values for positions plotted against ln[IC_50_] levels reveal a stronger, more statistically significant correlation with r= −0.83 (p= 8 × 10^−5^) for all modified sequences at position g2 (Figure 6B) while no statistically significant correlation is observed at position g4 (Figure 6C), a seed region C2′-OMe-modified sugar position we included here as a contrast to the g2 and g6 positions that both contain C2′-F modified sugars. Yet, at position g6, a fair correlation between ln[IC_50_] and $\Delta E_{6}$ with r= −0.62 (p= 7 × 10^−3^) is observed (Figure 6D). The nucleobase composition at positions g2–g8 of the seed region for all 15 modified sequences is summarized in Table S7. In contrast to many other positions, g2 especially, but also g6 are biased toward either A or U for the most active modified sequences, which correlates with lower average magnitude negative $\Delta E_{i}$ values compared with G or C bases (Figure 5A). To further investigate the unique character of position g2, we calculated the positional $\Delta E_{i}$ values using the NN model reviewed in the supplemental information. In Figure S7, the $\Delta E_{i}$ values for the first four positions g1–g4 are compared for each modified sequence. As expected, both active and inactive siRNAs exhibit low (less negative) $\Delta E_{1}$ values at position g1. For position g2, six out of the eight active siRNAs exhibit low (less negative) $\Delta E_{2}$ values, while all seven inactive siRNAs have high magnitude (more negative) $\Delta E_{2}$ values (−9 to −14 kcal/mol). Position g3 also trends toward lower (less negative) $\Delta E_{3}$ values for the active siRNAs, while there is no significant difference between active and inactive sequences at position g4. The significance of position g2 is also supported by the statistical model predicting ln[IC_50_], where the predicted $T_{m}$’s of the stacks that include that position were identified as the second and third most highly weighted features (Figure S6). Since g2 has the most pronounced preference for less negative $\Delta E_{i}$ values, we chose to focus on this position in this NN-based analysis (Figure S7).

### Position-specific sugar stereochemistry distributions, structures, and energies

It is well established for double-stranded RNAs that motions of the sugars are largely constrained to exist in the C3′-endo conformation.^44^ To address the question of how the chemical modifications (2′-OMe and 2′-F utilized for our modified siRNAs) may affect the sugar conformational preference, we examined the MD output for all positions for two pairs of siRNAs with opposite activity profiles for the modified sequences exhibiting either a substantial loss of activity or maintaining activity (siSER-1/siSER-1m and siSER11/siSER-11m), respectively. The number of instances of either C3′-endo or C2′-endo conformation observed across all positions are displayed in Figure S8. For the parent siSER-11, all positions exist in the C3′-endo conformation 100% of the time, while for the modified siSER-11m, 4% of the C2′-OMe and 7% of C2′-F modified sugars exist in the C2′-endo conformation. For the siRNA pair, which shows a loss of activity following chemical modification, the parent siSER-1 exhibits 2% of all sugars in the C2′-endo conformation while the siSER-1m features 5% of the C2′-OMe and 0% of 2′-F modified sugars in the C2′-endo conformation. These data suggest that modified duplexes generally possess an increased conformational flexibility over their parent counterparts, with variable levels exhibited at the duplex end positions of both parent and modified, but greater flexibility exhibited at modified sequences’ internal positions, predominantly at the C2′-F containing positions g2 and g6, as we develop in the following data. Next, we examined the MD simulation output in a position-specific manner for the two pairs of siRNAs (Table S8) tabulating the % time spent in the C2′-endo and C3′-endo conformations. For both parent siSER-11 and siSER-1 sequences, all internal positions were found to be in the C3′-endo conformation with a small % in C2′-endo at the terminal positions. Similarly, for the inactive siSER-1m, internal positions are predominantly in the C3′-endo conformation, although both terminal positions exhibit a substantial % of C2′-endo sugar pucker. In contrast, for the more active sequence siSER-11m, the internal positions g2 (C2′-F) and g13 were found to exist to a significant extent in the C2′-endo conformation (57% and 34%, respectively).

**Figure 7:**
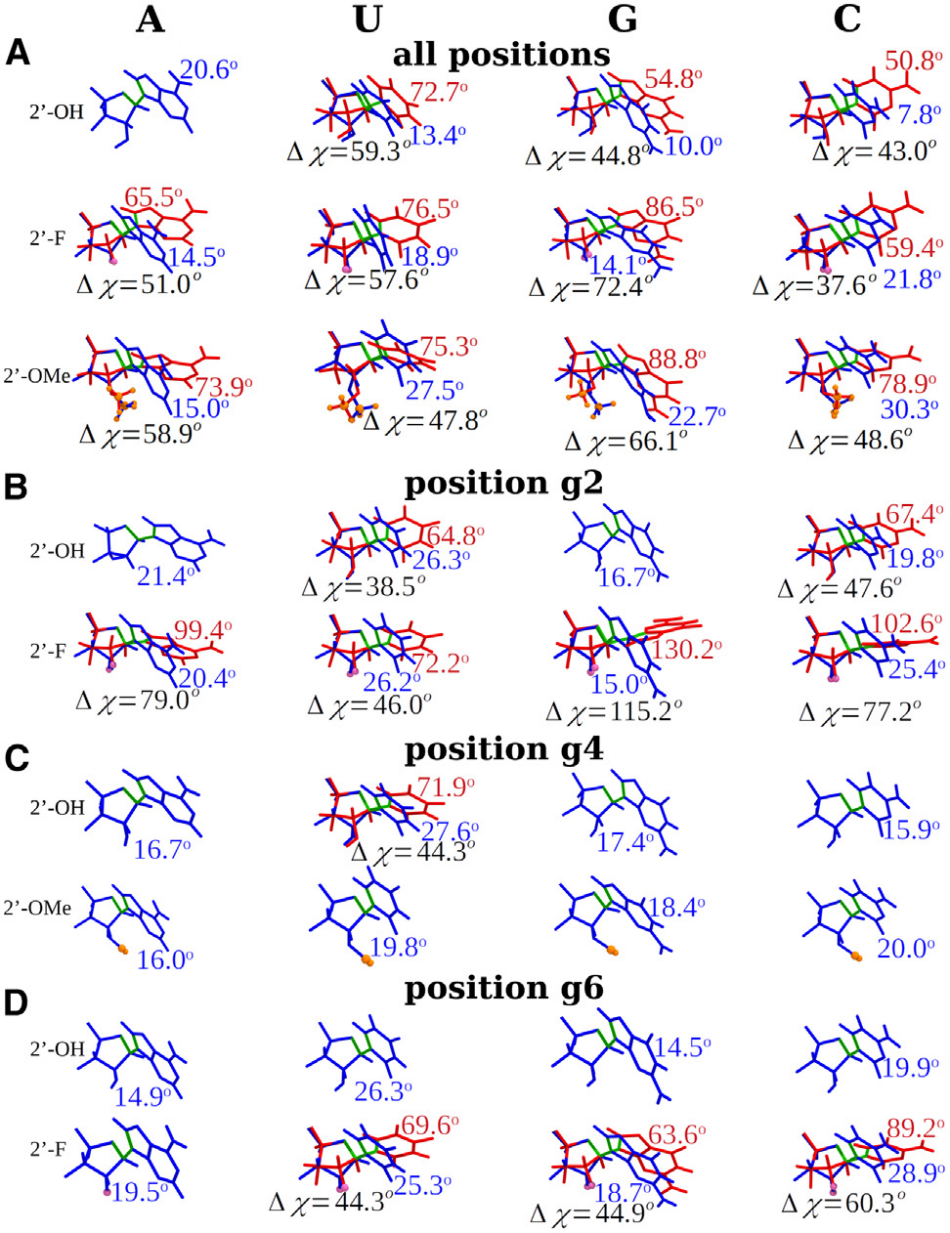
Structures of the nucleotides A, U, G, and C in C2′-endo and C3′-endo conformations Shown are structures for all siRNAs and at all positions (A) of A, U, G, and C nucleotides, as well as the average structures at positions g2 (B), g4 (C), and g6 (D) found in conformation C2′-endo (red) and C3′-endo (blue). For the most representative structures (A), parent nucleotides and nucleotides with 2′-OMe and 2′-F modifications are presented. For 2′-F modified positions g2 and g6 average structures (B and D), both parent and modified nucleotides are displayed, and for 2′-OMe position g4 average structures (C), both parent and modified nucleotides are shown. The χ dihedral angle (O4′-C1′-N-C1) is shown in green (light green for C2′-endo and dark green for C3′-endo). The values of χ are given for both C2′-endo (red) and C3′-endo (blue); the difference Δχ between C2′-endo and C3′-endo is shown in black.

From the perspective of the sugar stereochemistry distributions of individual modified sequences (Figure S8) and their position-specific properties (Table S8), we analyzed the average positional behavior of all 15 modified sequences for the four bases across the duplexes and for the seed region positions g2 (2′-F) and g4 (2′-OMe) of the guide strand (Table S9; Figure 7). For each position and base type, the number of instances and C2′-endo % of the total, as well as the energies of the stereochemical conformers, $\Delta E_{C2^{\prime }}$ and $\Delta E_{C3^{\prime }}$, and the energy difference $\Delta \Delta E_{C2'-C3'}$ was calculated. For all three positions, uridine (U) has significantly lower energies, followed by A, then C and G. For position g2 across all modified sequences, the pyrimidines U and C exhibit a much larger fraction of C2′-endo (27% and 29%, respectively) compared with the two purines A and G. This is likely due to the lower $\Delta E_{C2^{\prime }}$ values of the former. In contrast, position g4 shows no instance of C2′-endo conformation.

To further explore the position- and base-dependent effect on the sugar conformation, we analyzed the average dihedral angles χ for both C2′-endo and C3′-endo conformations as well as the differences in dihedral angles Δχ between conformations. Sugar conformations for all parent and 2′-F and 2′-OMe-modified siRNAs across all positions as well as for guide strand positions g2 and g4 were analyzed and averaged separately for the individual bases (Figure 7). The parent sequences (Figure 7A) generally exhibit relatively consistent χ and Δχ values for the four bases across the different positions that are characteristic of double helical RNAs.^45^ At position g2, the parent RNA purine nucleotides A and G exhibit no sugar pucker transitions, while their 2′-F versions show very large χ and Δχ values (Figure 7B). For U, both parent and 2′-F modified sugars have very similar χ and Δχ values while for C, the 2′-F modification gives rise to significantly higher χ and Δχ values compared with the parent. In contrast, position g4 exhibits no C2′-endo to C3′-endo stereochemical flexibility in either the parent or 2′-OMe-modified sequences (Figure 7C) with the single exception of U for the parent sequences, while position 6 exhibits intermediate levels of stereochemical flexibility for the 2′-F modified sequences (Figure 7D). Taken together, a strong base-dependent difference in sugar conformation between parent and 2′-F-modified siRNAs is observed at position g2 (and less so for g6) particularly for nucleobases G and C, which exhibit large χ and Δχ values. This suggests a preference for U (and A) at these positions, resulting in lower C2′-endo interaction energy $\Delta E_{C2^{\prime }}$, lower stability, and increased sugar stereochemical flexibility, which correlates with increased activity levels (Figure 6B).

## Discussion

Notwithstanding several decades of research, it is not well understood how different chemical substitutions within the passenger and guide strands affect the ability of the siRNA to engage with the RISC complex and mediate target mRNA to generate the active form of RISC. RISC loading is a multistep process involving several proteins and requiring the removal of the passenger strand multiple consecutive steps and extensive conformational changes of the enzyme complex.^46^^,^^47^ The complexity of the siRNA-RISC interactions limits our ability to predict siRNA activity and to rationally design highly potent chemically modified siRNAs. As an important first step, the focus of this study has been to understand whether and how single positions influence the activity of chemically modified siRNAs.

To improve our understanding of the interplay between siRNA sequence and chemistry resulting in the observed sequence-dependent tolerance for chemical modifications, we combined experimental and computational studies using 15 pairs of siRNAs, each composed of a parent and a chemically modified duplex. The main objectives for this study were (1) to correlate the experimentally accessible measures of biological activity (IC_50_ data) and thermodynamic stability ($T_{m}$ data) with their molecular structural, dynamic, and thermodynamic properties accessible in the MD simulations, and (2) to identify a subset of molecular characteristics that could be used to predict the biological activity of modified siRNA duplexes. While all parent siRNAs show dose-dependent activity with well-defined IC_50_ values, the set covers a wide range of potencies with differences in IC_50_ spanning several orders of magnitude from sub-nanomolar to high nanomolar values. When we imposed a well-established and generally well-tolerated chemical modification pattern on these parent unmodified sequences, the impact ranged from modest improvements to substantial loss of activity. Notably, this set of parent siRNAs also covers a wide range of duplex stabilities with melting temperatures $T_{m}$ measured in the 40°C–75°C range. The introduction of chemical modifications generally increased the $T_{m}$ by an average of 10°.

To determine potential causes for the observed differential response in potency to the same chemical changes across the set and enable a position-specific structure, dynamics, and energetics-based interpretation of the experimental results, we turned to computational molecular modeling.^48^^,^^49^ Using the reported bsc0χOL3 force field, we carried out long MD simulations of siRNA duplexes composed of canonical RNA (2′-OH ribonucleotide) in aqueous solution at room temperature. We developed a force field, which accounts for 2′-F and 2′-OMe ribose modifications (Figure S1), to generate 1-μs long trajectories of the dynamics of all 15 pairs of parent and modified siRNA duplexes (∼85 μs of total simulation time).

The MD simulations revealed that the presence of the 2′-modifications increases the area of molecular surface exposed to solvent (SASA) by an average of ∼5% for all modified siRNAs compared with their parent counterparts. This increase is due to the preponderance of 2′-OMe residue positions in the modified siRNAs since the position-based analysis of simulation results showed that SASA values of 2′-OMe residue positions were higher, while those of 2′-F residue positions were lower than the parent residue values. The observed increase in SASA correlates with a similar increase in average solvation energy of ∼4%. Hence, while base pairing and base stacking interactions reinforcing the secondary structure of chemically modified vs. parent dsRNA duplexes are similar, chemically modified dsRNA molecules with this substitution pattern generally form more soluble structures than their parent dsRNA counterparts. Further analysis revealed that the presence of chemical modifications has a significant impact on the tertiary structures. We found that modified siRNAs are shorter, denser, and have greater volume than their corresponding parent sequences.

The modified siRNA duplexes display a higher thermal stability compared with their respective parent sequences with an average $T_{m}$ increase of 10°C. We also observe a strong correlation between the experimental $T_{m}$ and the calculated duplex interaction energy ΔE and an average increase in the computed ΔE for the modified siRNAs of about 9 kcal/mol. The duplex interaction energy values ΔE correlate well with values derived from the NN model for parent siRNAs, which validates the computational modeling approach. Although the NN model can be used to estimate parent ΔE values, it is less accurate for modified siRNAs. The analysis also indicates a strong correlation between the calculated ΔE values and experimentally determined activity with the inactive siRNAs clustering around higher $T_{m}$ and higher (more negative) ΔE values. This suggests that, generally, a higher duplex stability for the modified siRNAs is associated with a greater potency loss, which is in line with conclusions from previously reported non-MD simulation studies.^50^^,^^51^ Our results imply that the interaction energies (ΔE) calculated using the all-atom MD simulations may be used to predict the impact of 2′-modifications on duplex stability and potency. However, unlike global thermal stability measurements, MD simulation also allow for a more detailed analysis of the contributions of individual positions to the physicochemical and biological properties of modified siRNAs.

While the global SASA values only showed a moderate correlation with the relative activity of the modified siRNAs, narrowing the selection to the guide strand seed region (g1–g8) resulted in a stronger correlation between SASA and the impact of the chemical modifications on potency. This analysis suggested that it would be interesting to deconvolute the contributions of individual positions to the physicochemical and biological properties of modified siRNAs. Analyzing the interaction energy and SASA profiles across the entire duplex on a per base pair basis showed the greatest impact of $\Delta E_{i}$ on siRNA activity at position g2 and, to a lesser extent, at position g6 with a lower (less negative) interaction energy and hence greater flexibility at these positions resulting in more active siRNAs. These results suggest that the sequence (nucleobase) identity at the 2′-F sugar positions g2 and g6 are the most impactful for siRNA activity with A and U bases exhibiting lower base stacking and pairing energies. These results are in good alignment with previous studies reporting a strong preference for weaker thermal stability at the 5′-end of the guide strand relative to the 3′-end for optimal RISC loading of the correct (antisense) strand.^52^^,^^53^ Importantly, however, our findings highlight the position-dependent impact of chemical modifications since the overall increase of the thermal duplex stability of modified siRNAs cannot adequately explain the potency loss observed with a subset of chemically modified siRNAs.

Our MD simulation-based findings point to the importance of lower stability and greater flexibility due to lower (less negative) $\Delta E_{i}$ values at guide strand position g2 and to a lesser extent g6 as key characteristics of active modified sequences. Interestingly, g2 and g6 are among the few key positions within the siRNA, which have been previously identified as being particularly intolerant to bulky 2′-modifications.^39^^,^^54^^,^^55^ Position g2 has also been found to be sensitive to modifications, such as GNA,^56^ α-(L)-threofuranosyl nucleic acid (TNA),^57^ or altritol nucleic acid (ANA),^58^ which alter the thermal stability and/or duplex structure suggesting that the tight interactions between guide strand and Ago2 at this position leave little room for structural perturbations. In addition, our findings indicate that for fully 2′-F/OMe-modified siRNAs greater flexibility and lower binding energy at the g2 position are required to maintain the intrinsic potency of their unmodified parent duplexes. Although our specific chemical sugar modification pattern in siRNA sequences has not been studied within Ago2 MID domain complexes, the X-ray studies of other modified duplex siRNAs complexed with Ago2^55^ have provided position-specific structural insights that are in qualitative agreement with some of our observations. At the 5′-end of the guide strand, position g1 has been observed to exist in the C2′-endo conformation, regardless of the type of 2′-modification. In the complexes, there exists a tight turn in direction between position g1 and position g2, and for position g2 a steric constraint exists, imposed by the MID domain interaction, that allows for a C2′-F but not the bulkier C2′-OMe sugar modification. The turn between position g1 and position g2 may prevent efficient base stacking, thereby requiring a lower stacking energy between bases for the Ago2 complex to form. We observed just such a lower stacking energy for position g2 in the MD simulation studies with concomitant greater flexibility and significant ability to transition between C2′-endo and C3′-endo conformations for the more active modified siRNAs in our study. For position g6, the X-ray studies of the complexes^55^ revealed a kink in the siRNAs between position g6 and position g7, with an Ile-365 amino acid side chain acting as a wedge from the Ago2 MID domain, inserting into the minor groove, that causes base stacking disruption. This helical disruption may also be the reason why position g6 requires a smaller 2′-F sugar modification, rather than the bulkier 2′-OMe, due to steric constraints in the complex. The disruptive kink may also be responsible for the preference for lower stacking energy bases at this position, along with concomitant greater sugar flexibility and (C2′-endo conformation potential), being associated with more active siRNA sequences.

The observed positional preferences for greater flexibility in the guide strand for the active modified siRNAs motivated us to examine the impact of the 2′-modifications on the sugar conformations. For parent dsRNAs, it is well established that the sugars are largely constrained to exist in the C3′-endo conformation.^45^ In an NMR study of the 2′-OMe sugars in the HIV-1 transactivation response element RNA, these sugars were found to be energetically biased toward the C3′-endo conformation^59^ and 2′-F substitutions are also known to preferentially exist in a C3′-endo conformation.^60^ These findings notwithstanding, we, therefore, examined the sugar stereochemistry aspect in more detail that exists in our pattern of fully modified siRNAs.

The results showed no conformational variation at any 2′-OMe-modified position (all C3′-endo), but for 2′-F modified positions an interesting difference between active and inactive siRNAs was observed, particularly at position g2, with inactive modified siRNAs exhibiting almost exclusive C3′-endo conformation at this position, while active modified siRNAs showed a significantly greater fraction of the C2′-endo conformation. We could further delineate the sequence/nucleobase dependence by analyzing the entire set of 15 modified siRNAs sequence at positions g2, g4, and g6. At position g2 (and to a lesser extent at g6), uridine residues possess increased flexibility (due to the 2′-F sugars) to readily transition between C2′-endo and C3′-endo stereochemistry, facilitated by the very low U stabilization energies, $\Delta E_{C2^{\prime }}$ and $\Delta E_{C3^{\prime }}$. Interestingly, the dihedral angles are very similar for both parent and 2′-F modified uridine sugars, while all the other bases show significant differences between parent and 2′-modified residues at g2. Combined with higher (more negative) energies $\Delta E_{C2^{\prime }}$ and a low fraction of C2′-endo for G and C, their large χ and Δχ values represent structures that are energetically difficult to achieve for those bases at that position. A similar, albeit somewhat less pronounced, nucleobase-dependent behavior can be found for position g6. These results tie together the nature of the nucleobase, the conformational flexibility and interaction energies at position g2, which our study has shown to be a critical position for the activity of chemically modified siRNAs. It is tempting to speculate that the required flexibility at this position, which is known for its sensitivity to bulky 2′-modifications,^55^^,^^61^ may compensate for the overall higher thermal stability of modified siRNAs and promote successful Ago2 loading.

In summary, based on studies of 15 parent (unmodified) and 2′-modified siRNA pairs spanning a range of *in vitro* potencies and targeting two different transcripts, we have identified several molecular features that can explain their physicochemical and biological properties. We have been able to show that (1) the computed interaction energetics $\Delta E_{i}$ and SASA values for the entire siRNA duplexes are correlated with experimental IC_50_ and $T_{m}$ data; (2) the computed seed region SASA correlates well with the relative *in vitro potencies* observed for parent and modified siRNAs with the latter showing significantly higher SASA values; (3) for $\Delta E_{i}$ at individual positions, g2 shows the strongest correlation with biological activity followed by g6 (both 2′-F modified sequence positions); (4) nucleobases U and A, which exhibit significantly lower (less negative) $\Delta E_{i}$ values compared with G or C, dominate the most active sequences at position g2 suggesting that low stability and greater flexibility at g2 are requirements for chemically modified siRNAs to maintain the potency of their parent counterparts; and (5) conformational flexibility in the sugar pucker (C3′- to C2′-endo transitions) at position g2 (and less so at g6) appears to be associated with higher activity, which helps explain the large impact of sequence at these positions. Although based on a limited number of siRNA sequences, our findings offer the molecular-level insights into why some well-established 2′-modification patterns might fail, for certain sequences, to generate active molecules, and provide useful guidelines for the design of potent, fully modified siRNAs.

## Materials and methods

### Duplex design

The chemical modification pattern we used is composed of 2′-F modifications at the guide strand positions g2, g6, g14, and g16 and passenger strand positions g5, g7, g8, and g9 with the remaining positions modified with 2′-OMe (Table 1; Figure 1).^13^ A dTdT overhang was added to the 3′ end of both strands. This chemistry template was applied to each of the 15 duplex sequences and compared with the unmodified parent compounds (all RNA with 3′-dTdT overhangs).

### siRNA synthesis

All oligonucleotides were prepared on a MerMade 192 synthesizer on a 1-μM scale using universal supports. All phosphoramidites were used at a concentration of 100 mM in 100% acetonitrile or acetonitrile:DMF (9:1) with a standard protocol for 2-cyanoethyl phosphoramidites, except that the coupling time was extended to 400 s. Oxidation of the newly formed linkages was achieved using a solution of 50 mM iodine in acetonitrile:water (9:1) to create phosphate linkages. After the synthesis (DMT-off), columns were incubated with 150 μL of 40% aqueous methylamine for 30 min and the solution drained via vacuum into a 96-well plate. After repeating the incubation and draining with a fresh portion of aqueous methylamine (150 μL), the plate containing crude oligonucleotide solution was sealed and shaken at room temperature for an additional 60 min to completely remove all exocyclic and cyanoethyl protecting groups. For RNA-containing oligonucleotides, 200 μL of DMSO and 300 μL of triethylamine trihydrofluoride were added followed by heating to 60°C for 60 min to remove all silyl protecting groups. Precipitation of the crude oligonucleotides was accomplished via the addition of 1.2 mL of 9:1 acetonitrile:EtOH (conjugates) or 1:1 EtOH:iPrOH (RNA) to each well followed by centrifugation at 3,000 rpm for 45 min, the supernatant removed from each well, and the pellets resuspended in 950 μL of 20 mM aqueous NaOAc. Oligonucleotides were purified using anion exchange chromatography (IEX) over a GE Source 15Q column (4.6 × 100 mm) with a linear gradient from 100 to 600 mM NaBr over 20 min in 20 mM sodium phosphate buffer (pH 8.5) with 15% acetonitrile at 60°C. The desired fractions were desalted over a GE Hi-Trap Desalting Column (Sephadex G25 Superfine) using water to elute the final oligonucleotide products. All identities and purities were confirmed using ESI-MS and IEX HPLC, respectively.

### Measurements of melting temperature

The melting studies were performed in 1-cm path length quartz cells on a Cary 300 spectrophotometer equipped with a thermoprogrammer. siRNA duplexes were evaluated at a duplex concentration of 2 μM in 1× PBS (10 mM Na*/*K phosphate buffer, pH 7.4, with 137 mM NaCl and 3 mM KCl). Each cuvette contained 800 μL of sample solution covered by 200 μL of light mineral oil. Melting curves were monitored at 260 nm with a heating rate of 1°C*/*min from 15°C to 90°C. Melting temperatures ($T_{m}$) were calculated from the first derivatives of the heating curves and the reported values are the result of at least two independent measurements.

### Measurements of RNAi potency *in vitro*

siRNAs targeting *SERPINA6* and *AGT* were evaluated for their gene silencing activity (IC_50_) in cell culture. Hep3B cells (ATCC) were transfected in a 384-well format with Lipofectamine RNAiMax and siRNAs diluted in Opti-MEM to create an 11-point (10-fold dilution steps) dose-response curve between 100 nM and 0.01 fM. Cells were incubated for 24 h at 37°C and then lysed for RNA isolation. An automated protocol on a BioTek-EL406 platform using DYNABEADS (Invitrogen, cat #61012) was used, the RNA bound to beads was washed twice with Wash Buffer A and once with Wash Buffer B. The beads were washed once with elution buffer and the supernatant removed. A cDNA mixture was added to the beads to elute RNA. Plates were sealed, mixed, and incubated for 10 min on an electromagnetic shaker at room temperature followed by 2 h at 37°C (Applied Biosystems High Capacity). Lightcycler 480 master mix (Roche, cat #04887301001) was mixed with GAPDH Taqman VIC probe and gene specific FAM probe was used to determine gene expression and measured on a LightCycler 480 Read Time PCR system (Roche) (Taqman probes: Hs00163881, Hs01586213, Hs02547823). Each duplex was tested in quadruplicate and data were normalized to cells transfected with PBS mock. To calculate relative fold change, the ΔΔCt method was used and IC_50_ values were calculated with Excel software extension XLFit version 5.3.1.3.^62^

### Force field parameters for all-atom MD simulations

Atomic partial charges for modified siRNA duplexes were calculated as described in the supplemental information. The atom types for atoms in the chemically modified ribose rings were determined using the Antechamber package.^63^ The molecular mechanics parameters, such as the equilibrium bond distances ($r_{0}$), spring constants for covalent bonds ($k_{b}$), equilibrium bond angles ($\theta_{0}$), and spring constants for bending of bond angles ($k_{\theta }$), as well as torsional angle parameters, including the magnitude associated with torsion energy ($V_{n}/2$), phase offset (γ), and periodicity (n), were obtained from the bsc0_χ_OL3 force field for the atom types described therein (except for the F-atom) as described in our previous study.^64^ Parameters related to the F-atom at 2′ position and its connections with ribose (i.e., covalent bonds, bond angles, and dihedral angles) were derived from the general AMBER force field GAFF^65^ (Tables S1 and S2).

### MD simulations and data analysis

siRNA duplex structures were constructed as described in the supplemental information. The force field parameters for 2′-F and 2′-OMe groups were obtained from the general AMBER force field (GAFF)^65^ (Tables S1 and S2). Similar to our previous MD studies on dsRNAs,^66^^,^^67^ using TIP3P water molecules, each siRNA duplex system was solvated up to 15Å from any of the solute atoms. The number of water molecules included in the octahedral solvation box of volume 450 nm^3^ was ∼10^4^. First, each siRNA duplex (RNA plus counterions plus water molecules) was energy-minimized to eliminate possible steric clashes for 10,000 steps of the steepest descent (water molecules and counterions), and then for 5,000 steps of the conjugate gradient with 50 kcal/mol restraint on the solute atoms. Next, each siRNA duplex system was heated from 0 K to 300 K over 50 ps time. Solvent molecules were relaxed using short MD simulations (250 ps) with 30 kcal/mol restraint on the solute atoms at T= 300 K temperature. The equilibration step involved 100 ps of restrained (0.05 kcal/mol on solute atoms) MD simulations. The MD simulations for all siRNA duplexes were carried out using the periodic boundary conditions.^31^^,^^68^ The 1-μs-long unrestrained equilibrium MD simulations for all siRNA duplexes were performed in the NPT ensemble using the CUDA version of pmemd^68^ in conjunction with the GPU accelerated version^69^^,^^70^ of AMBER 20.^71^ The particle mesh Ewald method was utilized to calculate the long-range electrostatics with the 10Å-cutoff. SHAKE was used to treat light hydrogen atoms. The equilibrium MD simulations for all siRNA duplexes were carried out at T= 300 K temperature using the Δt= 2 fs integration step; the 1-atm constant pressure was maintained using a Berendsen barostat with the 1-ps time constant.^72^ The 300 K constant temperature was maintained using a Berendsen thermostat with the 4-ps time constant. Numerical output from the all-atom MD simulations at equilibrium (last 750-ns part of the 1-μs trajectory) for each siRNA duplex was analyzed as described in the supplemental information. Analyses of the output from the MD simulations, including hydrogen bonds, base pairing, and base stacking interactions, as well as calculation of the thermodynamic state functions were carried out as described in the supplemental information.

## Data and code availability

All data are available from the corresponding authors upon reasonable request and included in the main text and supplemental material.

## Acknowledgments

V.B. and K.A.M. would like to acknowledge funding from 10.13039/100006400Alnylam Pharmaceuticals.

## Author contributions

E.K. and F.M. performed the MD simulations and analyzed the data. J.Z. performed the statistical modeling. S.H. and A.C. designed the siRNAs and generated the *in vitro* potency data. S.W. synthesized the siRNAs. M.K.S. measured the siRNA thermal stability. E.K., F.M., S.H., J.Z., K.A.M., M.A.M., and V.B. conceptualized and designed the studies, analyzed, and interpreted the data, and wrote the manuscript with input from all authors.

## Declaration of interests

J.Z., S.H., S.W., A.C., M.K.S., and M.A.M. are, or were during the time this work was conducted, employees of Alnylam Pharmaceuticals.

## Supplemental information

Document S1. Figures S1–S8 and Tables S1–S9 and Supplemental methods

Document S2. Article plus supplemental information
