# Supplementary material for: Improving the potency prediction for chemically modified siRNAs through insights from molecular modeling of individual sequence positions
Source: Mol Ther Nucleic Acids. 2024 Dec 5;36(1):102415. doi: 10.1016/j.omtn.2024.102415 (PMC11960531; doi:10.1016/j.omtn.2024.102415)
Supplement: Document S1. Figures S1–S8 and Tables S1–S9 and Supplemental methods [file mmc1.pdf]

OMTN, Volume *36*

Supplemental information

Improving the potency prediction for chemically modified siRNAs through insights from molecular modeling of individual sequence positions

Evgenii Kliuchnikov, Farkhad Maksudov, Jeffrey Zuber, Sarah Hyde, Adam Castoreno, Scott Waldron, Mark K. Schlegel, Kenneth A. Marx, Martin A. Maier, and Valeri Barsegov

Supplemental Figures

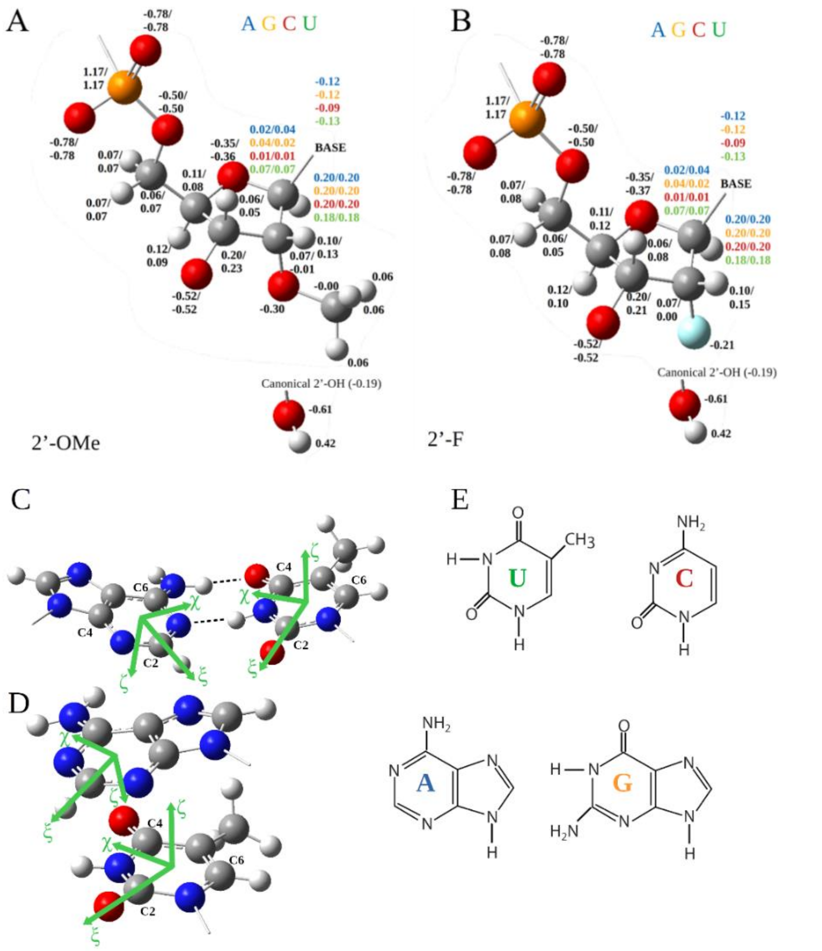

Figure S1. Atomic partial charges for backbone structure, and base pairing and base stacking interactions in siRNAs: Panels **A** and **B**: Shown are the energy-minimized structures and RESP charges for a fragment of RNA backbone with chemical ribose modification 2′-OMe (panel **A**) and 2′-F (panel **B**). The calculated atomic partial charges for modified RNA are compared with the charges on the same atoms for parent RNA (with 2′-OH group) extracted from bsc0χOL3 force field and displayed as a reference (separated by a slash). The total charge is shown in a different color for each base: adenine (A; blue), cytosine (C; red), guanine (G; orange), and uracil (U; green). The ribose ring and phosphate group are shown in the C3′-endo conformation without bases for clarity. Panel **C**: Local coordinate systems for purines and pyrimidines are used to describe the base pairing interactions. The center of the base ring atoms C2, C4, and C6 represents the origin of the local coordinate system as shown. The ξ-and χ-axes lie in the plane of the base while the ζ-axis lies normal to the $\xi\chi$-plane. The ξ-axis is pointed in the C2-atom direction, and the χ-axis is pointed toward the C4-atom (for C and U) or toward the C6-atom (for A and G). The two bases are forming a base pair via hydrogen bonds represented as dashed black lines. Panel **D**: Local coordinate system used for describing the base stacking interactions. The local coordinate systems (in panels **C** and **D**) are different from the coordinate system associate with the dsRNA duplex (see x-, y-, and z-axes in Fig. S2). Panel **E**: Structures of four ribonucleic acid bases: adenine (A; blue), cytosine (C; red), guanine (G; orange), and uracil (U; green).

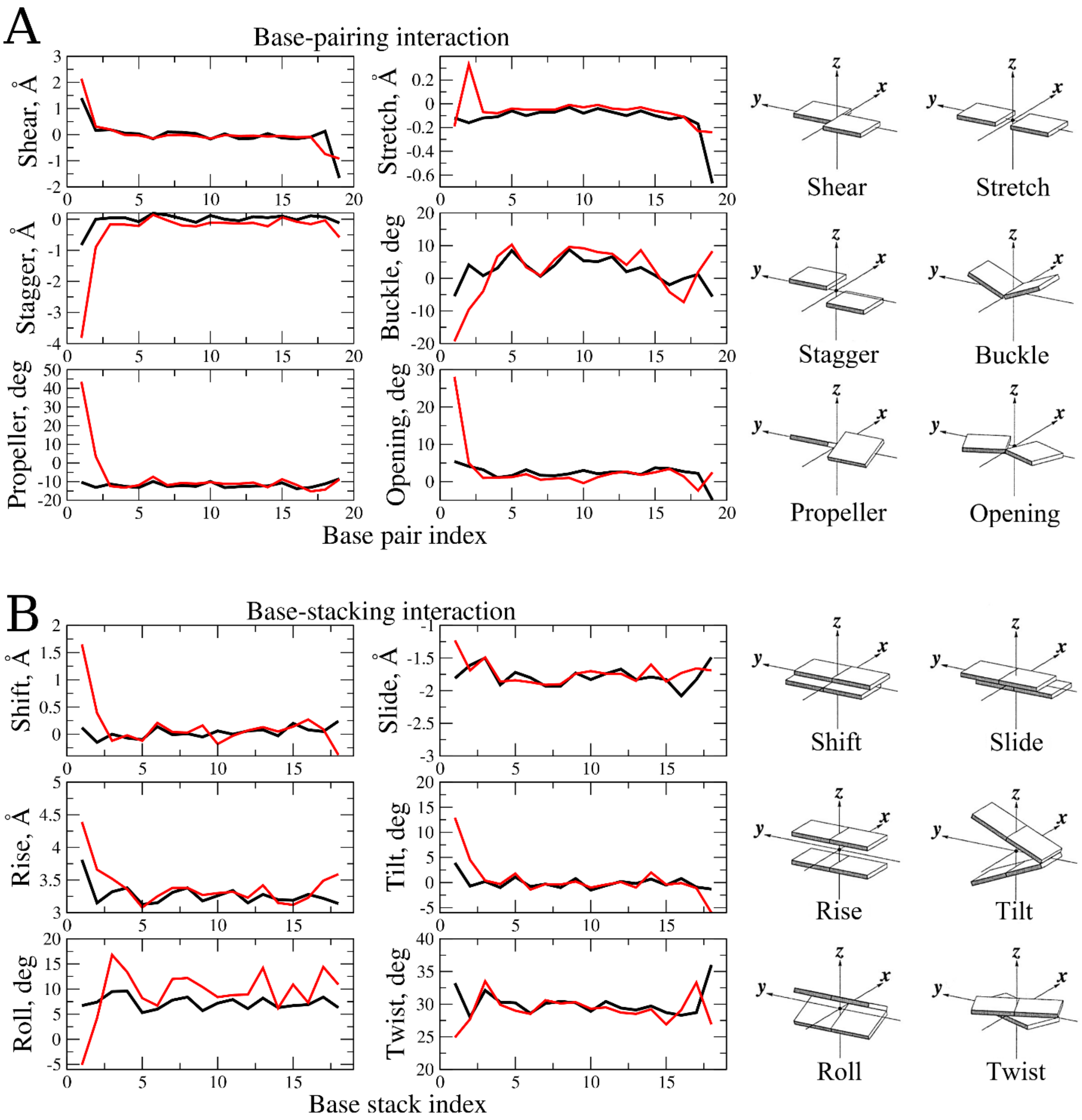

Figure S2. Helical properties of siRNA duplexes siSER-10 and siSER-10m: Base-pairing interaction parameters (shear, stretch, stagger, buckle, propeller, and opening) for each base pair (panel **A**) and base-stacking interaction parameters (shift, slide, rise, tilt, roll, and twist) for each base stack in the guide strand (panel **B**) for parent siRNA duplex siSER-10 (black curves) and siSER-10m (red curves).

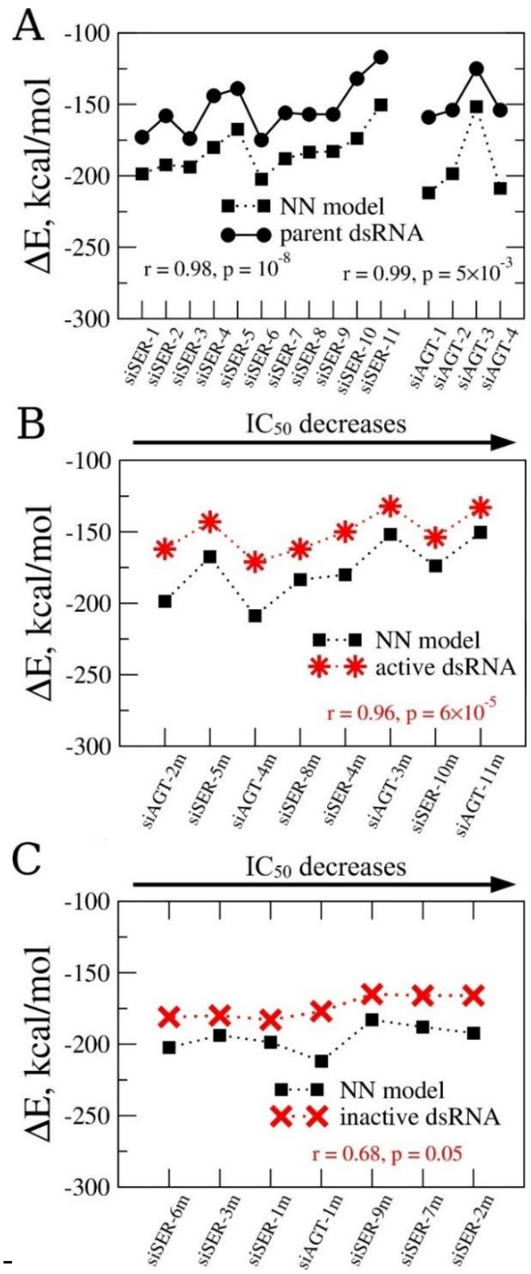

Figure S3. Thermodynamic state functions for siRNAs from MD simulations and Nearest Neighbor model calculations: Profiles of the interaction energies ΔE for all the parent siRNAs (panel **A**) and for the modified siRNAs (panels **B** and **C**) obtained from the MD simulations are compared with the profiles of ΔE calculated using the empirical NN model (black squares connected by dashed lines). Data for all the parent siRNA are displayed in black circles. Data for the modified siRNAs are categorized by their IC_{50} values as active (red stars in panel **B**), and inactive (red crosses in Panel **C**). In panels **B** and **C**, siRNAs are ordered (left to right) by the decreasing values of their IC_{50} from highest to lowest. Also shown are numerical values of the Pearson correlation coefficient r and the p-values calculated for correlations between the NN model and the parent siRNA, between the NN model and the active siRNAs, and between the NN model and inactive siRNAs.

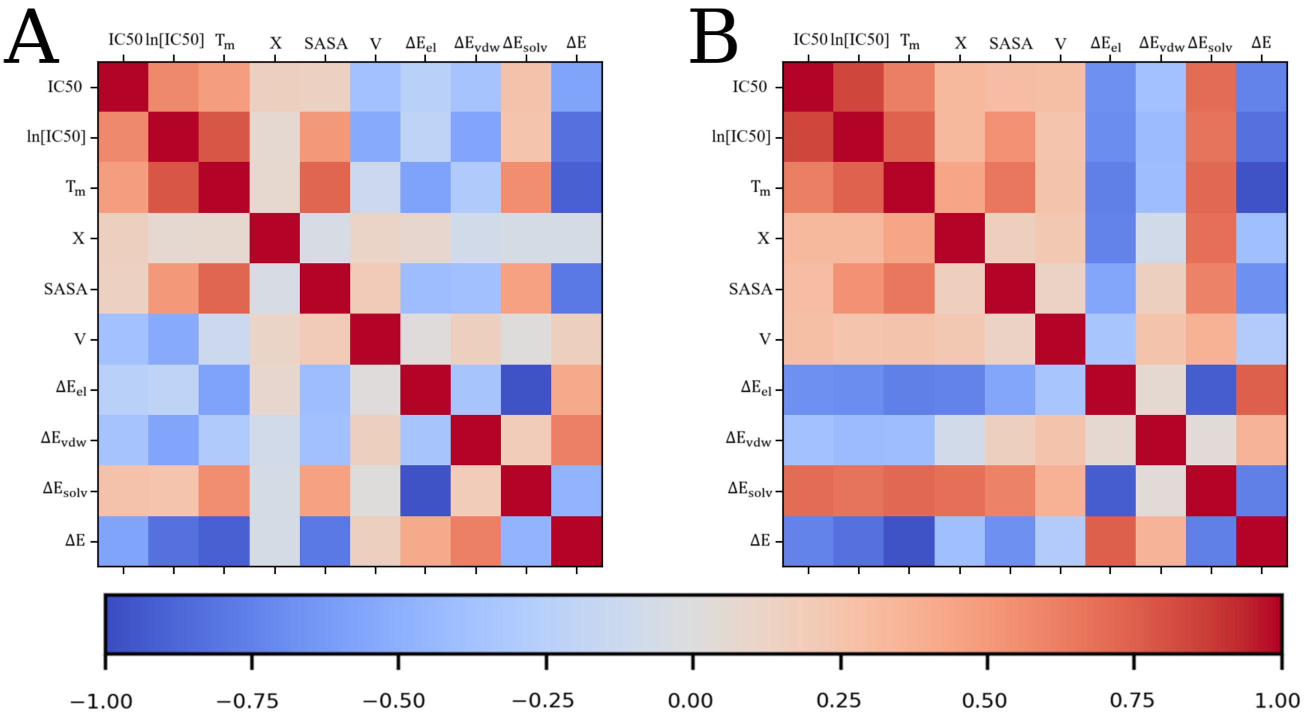

Figure S4. Maps of correlations between experimental IC_{50} and T_m data and theoretical quantities accessible from MD simulations: The heat maps were generated based on all 15 pairs of siRNA duplexes. Features (input variables) for the correlation analysis were taken from Tables S3, S4. The values of Pearson correlation coefficient were calculated for parent siRNAs (panel **A**), and for modified siRNAs (panel **B**). The color bar sets the correlation amplitude.

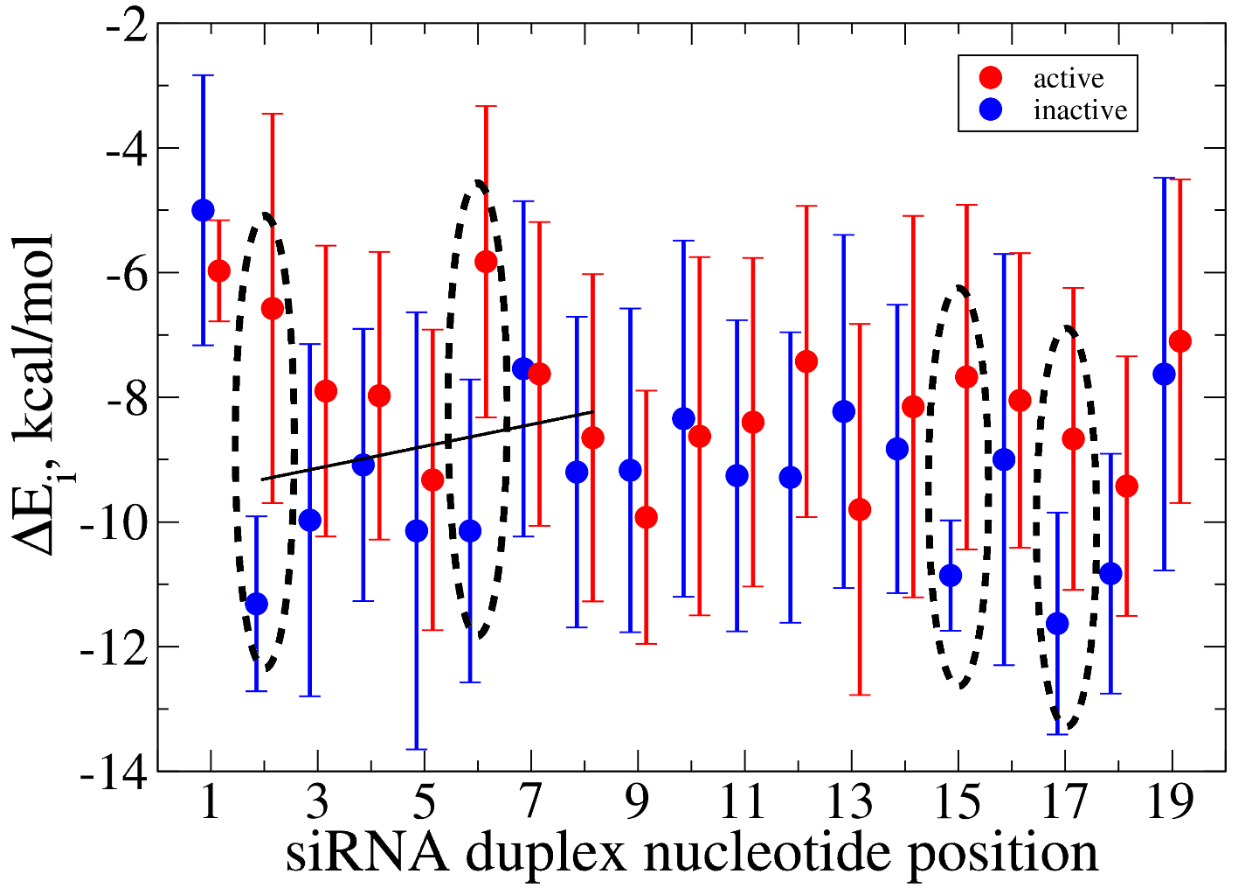

Figure S5. Position dependent profile of interaction energy per base pair: Shown are the profiles of the average ΔE_i and standard deviation for modified siRNA vs. the position of the nucleotide (guide positions g1-g19), for the active (red circles) and inactive (blue circles) modified siRNAs, calculated based on the output from the MD simulations for all 15 siRNA sequences. Structures were obtained in the presence of base pairing and base stacking from double helices. Black line shows the linear SVM (support vector machine) model-based separation between the more active and less active siRNAs in the seed region only (guide positions g1-g8). Positions with the biggest separation between the inactive and active siRNAs are circled with black dashed lines.

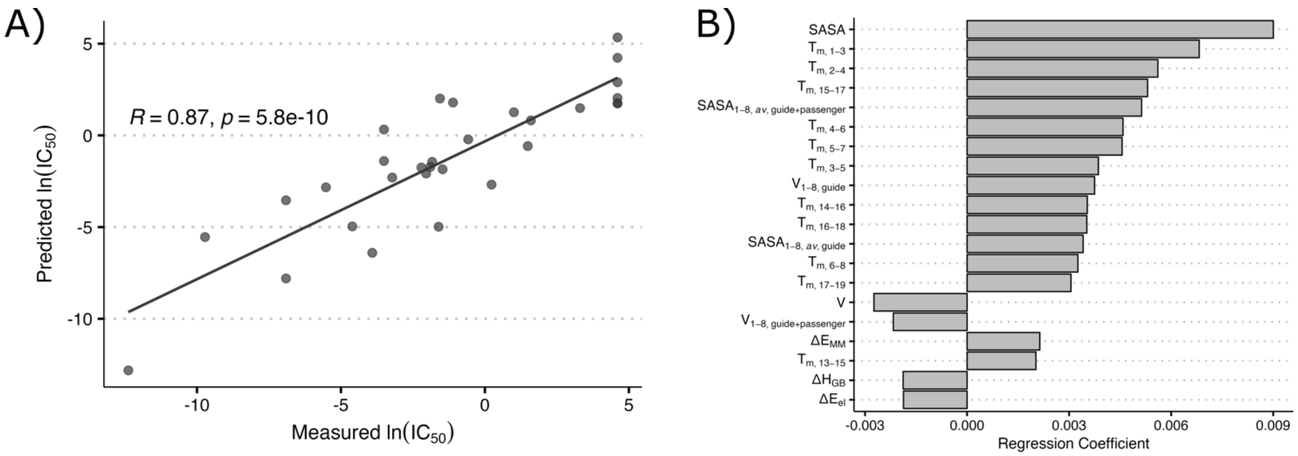

Figure S6. Machine Learning based determination of relative importance of model features: Panel **A**: Performance of the PLS model after omitting features related to the geometry of base pairs. A three-component PLS model was fit to the rescaled log-transformed IC_{50} values for the entire set of siRNAs. The model resulted in an $MSE = 4{,}66$ and $R = 0.87$. Predicted IC_{50} plotted against observed IC_{50} measurements. The MSE for the LOOCV is 8.23. Panel **B**: The regression coefficients obtained from the three-component PLS model. The component weights were mapped to the feature space to generate an equivalent linear model. The top 20 features are shown.

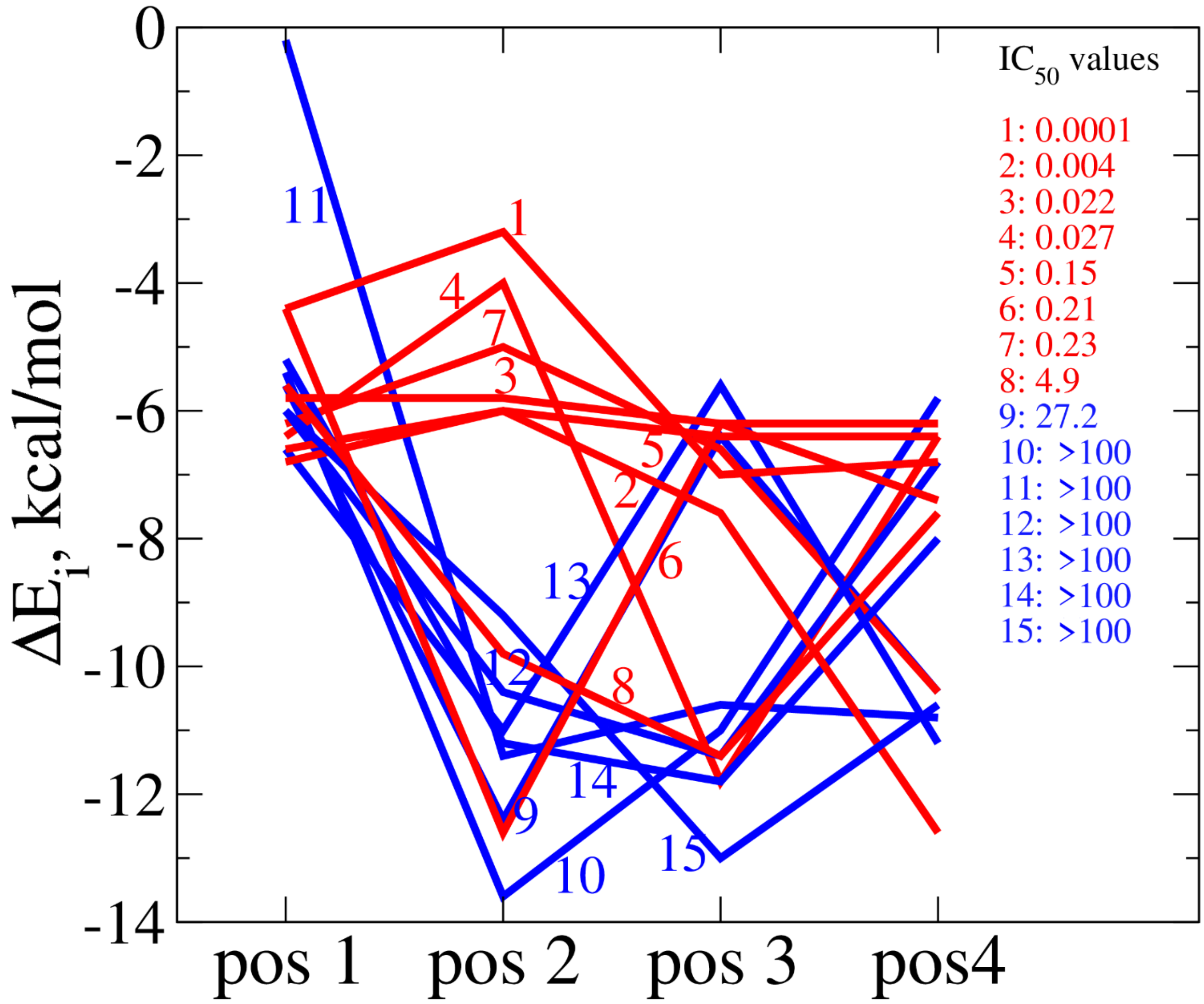

Figure S7. Per-nucleotide g1-g4 interaction energy decomposition and statistics of activity change after sugar modifications: Profiles of the interaction energy ΔE_i per base pair for the guide strand nucleotides i = g1-g4 for all modified siRNAs, active (red) and inactive (blue). Each line is numbered from 1 (the most active) to 15 (the least active); for each number, the value of IC_{50} is given in nM.

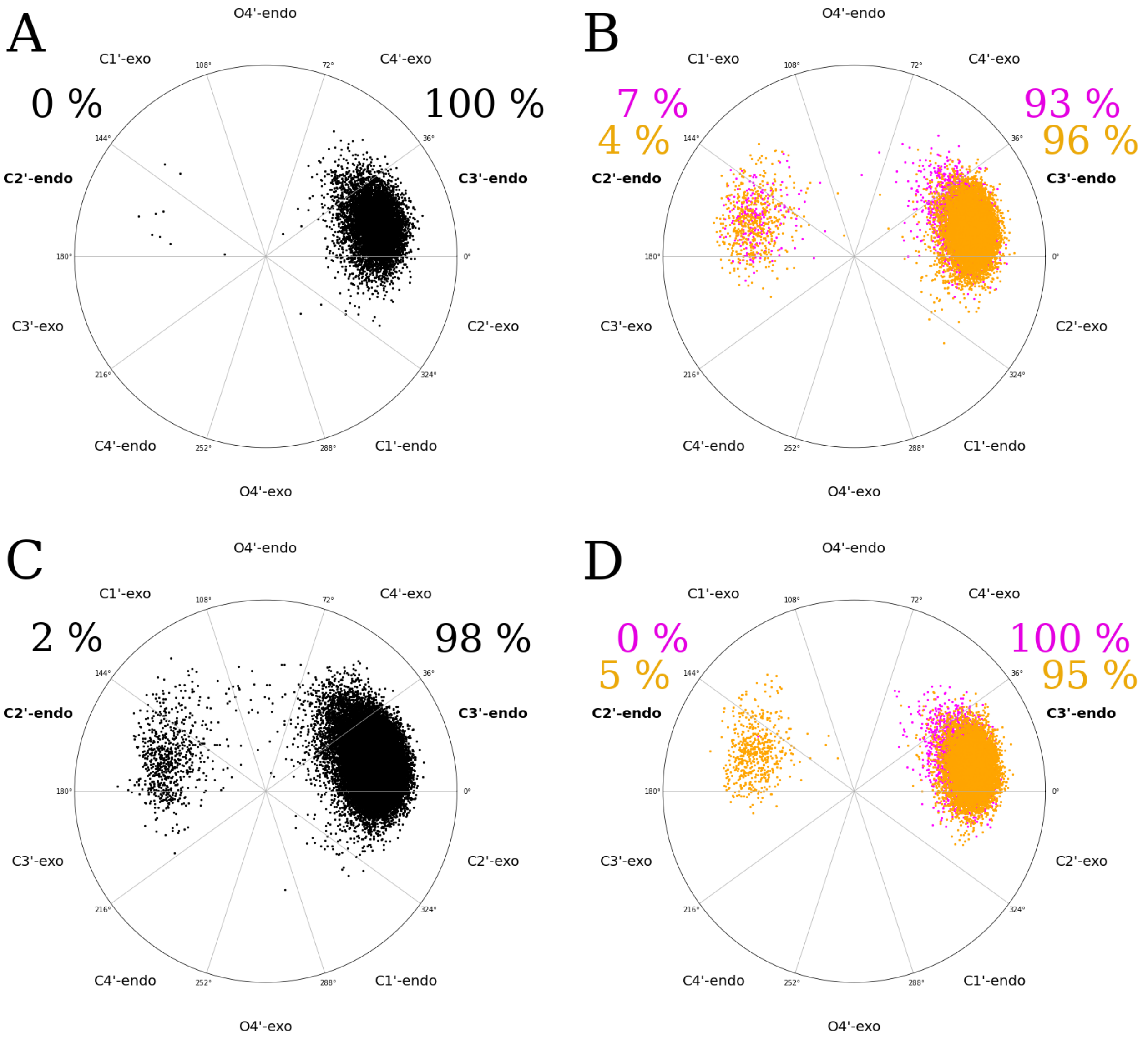

Figure S8. Distribution of pseudo-rotation phase angles of the ribose ring for siRNA duplexes: Values of the phase angle *P* given in multiples of 36° (based on Altona-Sundaralingam definition; see Ref. [1]) showing the sugar pucker states on the periphery of the circle. Panels **A** and **B**: shown are the sugar pucker states for siSER-11 and SER11m, respectively. Panels **C** and **D**: shown are the sugar pucker states for siSER-1 and siSER-1m, respectively. In each panel we show the analysis of the MD output classifying the number of instances (points) of either C3′-endo or C2′-endo configuration observed at all positions. Each data point represents a single nucleotide at a particular time instant. Different colors represent various sugar chemistries: parent ribose (2′-OH; black circles), 2′-F modification (magenta circles), and 2′-OMe modification (orange circles).

Supplemental Tables

Table S1. Molecular Mechanics parameters for covalent bonds and bond angles for chemically modified ribose ring in siRNA molecules: Shown for each covalent bond are the equilibrium bond distance r_0 and spring constant k_b, and for each bond angle are the equilibrium bond angle θ_0 and bond angle spring constant k_a. These force field parameters for 2′-OMe and 2′-F modifications in the ribose ring were determined for the modified siRNA molecules (see Fig. 1 in main text). The following atom types are described: CT – sp^3 hybridized carbon with 4 explicit substituents; F – fluorine atom; H1 – hydrogen on aliphatic carbon with 1 electron-withdrawing group; and OS – ester oxygen.

Bond length	k_b, kcal·mol^{-1}·Å^{-2}	r_0, Å
CT-F	367	1.38
Bond angle	k_a, kcal·mol^{-1}·rad^{-2}	θ_0, deg
OS-CT-H1	50	109.5
CT-OS-CT	60	109.5
F-CT-CT	50	109.0

Table S2. Molecular Mechanical parameters for torsion angles for modified ribose ring in siRNAs: Shown for each torsion angle are the number of bond paths, the magnitude of torsion energy $V_n/2$, the phase offset γ and the torsion periodicity n. These force field parameters for 2′-OMe and 2′-F substitutions in the ribose ring were determined for modified siRNA duplexes explored in this study (see Table 1 and Fig. 1 in main text). Atom types are described in Table S1.

Torsion angle	no. of paths	$V_n/2$, kcal/mol	γ, deg	n
H1–CT–CT–F	1	0.19	0	1
CT–CT–CT–F	1	1.10	180	2
N*–CT–CT–F	9	1.40	0	3
OS–CT–CT–F	9	1.40	0	3

Table S3. Statistics of molecular properties of siRNA duplexes: Accumulated for each canonical (first entry) and modified (second entry) siRNA duplex are: the end-to-end distance X, solvent accessible surface area (SASA), molecular volume V, molecular density ρ, and root mean square deviation (RMSD). For X, SASA, and RMSD the average values and standard deviations are shown). Black color represents the parent siRNAs, siRNA sequences inked in blue are categorized as siRNAs that lose activity after the modification ("inactive"), and siRNA sequences inked in red are categorized as siRNAs that retain their activity in the presence of 2′modifications ("active").

siRNA code	X, nm	SASA, Å^2	V, Å^3	ρ, g/ml	RMSD, nm
siSER-1 / siSER-1m	5.71±0.16/ 5.48±0.15	7,582±107/ 7,954±70	8,765/ 9,594	2.525/ 2.383	0.62±0.08/ 0.55±0.05
siSER-2 / siSER-2m	5.69±0.23/ 5.48±0.19	7,643±82/ 7,998±1047	8,949/ 9,253	2.468/ 2.465	0.40±0.06/ 0.46±0.05
siSER-3 / siSER-3m	5.65±0.27/ 5.43±0.22	7,661±62/ 8,070±1197	8,988/ 9,499	2.462/ 2.406	0.31±0.07/ 0.45±0.17
siSER-4 / siSER-4m	5.75±0.26/ 5.47±0.16	7,608±97/ 8,019±83	8,848/ 9,276	2.487/ 2.451	0.39±0.08/ 0.36±0.07
siSER-5 / siSER-5m	5.71±0.23/ 5.51±0.19	7,514±68/ 7,898±108	9,001/ 9,332	2.442/ 2.433	0.31±0.06/ 0.33±0.06
siSER-6 / siSER-6m	5.72±0.26/ 5.61±0.19	7,696±77/ 8,010±71	8,968/ 9,363	2.468/ 2.441	0.34±0.06/ 0.40±0.07
siSER-7 / siSER-7m	5.39±0.19/ 5.49±0.14	7,604±70/ 7,921±85	8,632/ 9,505	2.555/ 2.397	0.34±0.08/ 0.38±0.08
siSER-8 / siSER-8m	5.99±0.20/ 5.53±0.23	7,568±86/ 7,996±70	8,634/ 10,140	2.555/ 2.247	0.47±0.08/ 0.27±0.05
siSER-9 / siSER-9m	5.66±0.24/ 5.55±0.16	7,647±68/ 8,018±91	9,502/ 9,503	2.332/ 2.397	0.33±0.06/ 0.34±0.06
siSER-10 / siSER-10m	5.63±0.29/ 5.44±0.21	7,529±62/ 7,950±91	8,954/ 9,304	2.458/ 2.443	0.38±0.05/ 0.40±0.05
siSER-11 / siSER-11m	5.65±0.24/ 5.50±0.18	7,511±53/ 7,830±87	8,926/ 9,268	2.457/ 2.445	0.50±0.08/ 0.43±0.07
siAGT-1 / siAGT-1m	5.70±0.16/ 5.55±0.15	7,697±75/ 8,002±79	10,362/ 9,816	2.136/ 2.329	0.24±0.06/ 0.30±0.05
siAGT-2 / siAGT-2m	5.71±0.16/ 5.49±0.13	7,628±91/ 7,996±86	9,374/ 9,213	2.353/ 2.473	0.39±0.09/ 0.30±0.06
siAGT-3 / siAGT-3m	5.72±0.15/ 5.42±0.14	7,530±87/ 7,928±121	10,244/ 9,448	2.139/ 2.396	0.33±0.07/ 0.32±0.07
siAGT-4 / siAGT-4m	5.77±0.22/ 5.53±0.15	7,608±77/ 8,053±89	10,224/ 9,252	2.162/ 2.468	0.23±0.05/ 0.34±0.07
Average parent / modified	5.70±0.12/ 5.50±0.05	7602±62/ 7976±63	9225±591/ 9451±251	2.340±0.145/ 2.412±0.060	0.37±0.11/ 0.40±0.13

Table S4. Thermodynamic molecular properties quantifying interactions between the guide and passenger strands comprising siRNA duplex: Accumulated for each parent (first entry) and modified (second entry) siRNA duplex are the values of components of the interaction energy between the guide and passenger strands (averages and standard deviations): electrostatic energy ΔE_{el}; van der Waals energy ΔE_{vdW}; and solvation energy ΔE_{solv}. Also shown are the values of interaction energy ΔE; interaction entropy $T\Delta S$; and interaction free energy ΔG. Black color represents the parent siRNAs, siRNAs inked in blue are categorized as sequences that lose activity and those inked in red are categorized as sequences that retain their activity in the presence of 2′-modifications.

siRNA code	ΔE_{el}, kcal/mol	ΔE_{vdW}, kcal/mol	ΔE_{solv}, kcal/mol	ΔE, kcal/mol	$T\Delta S$, kcal/mol	ΔG, kcal/mol
siSER-1 / siSER-1m	5710±158/ 5943±154	-129±7/ -140±10	-5755±155/ -5985±150	-173±8/ -182±8	-96±6/ -94±2	-77±6/ -88±5
siSER-2 / siSER-2m	5760±154/ 5975±183	-126±9/ -135±9	-5791±150/ -6005±177	-158±8/ -165±9	-95±4/ -91±2	-63±5/ -74±4
siSER-3 / siSER-3m	5668±151/ 5868±170	-124±7/ -131±8	-5718±148/ -5916±166	-174±7/ -179±8	-95±6/ -87±8	-79±5/ -92±7
siSER-4 / siSER-4m	5810±158/ 6026±159	-120±8/ -134±9	-5834±154/ -6040±176	-144±9/ -149±9	-93±5/ -88±4	-51±10/ -61±9
siSER-5 / siSER-5m	5929±152/ 5943±154	-127±7/ -136±11	-5941±150/ -6128±166	-139±6/ -142±8	-95±2/ -86±3	-44±6/ -56±8
siSER-6 / siSER-6m	5689±159/ 5776±163	-124±10/ -135±8	-5739±156/ -5820±158	-175±7/ -180±9	-94±4/ -86±2	-81±6/ -94±9
siSER-7 / siSER-7m	5731±151/ 5914±180	-128±9/ -138±8	-5758±147/ -5941±174	-156±8/ -165±9	-87±5/ -88±1	-69±8/ -77±4
siSER-8 / siSER-8m	5725±164/ 5887±139	-124±7/ -126±8	-5757±162/ -5919±137	-157±7/ -161±8	-92±5/ -87±2	-65±8/ -74±8
siSER-9 / siSER-9m	5701±146/ 5760±170	-121±8/ -127±8	-5737±144/ -5798±168	-157±7/ -164±8	-84±3/ -86±3	-73±4/ -78±6
siSER-10 / siSER-10m	5681±194/ 6062±241	-74±12/ -132±11	-5787±188/ -6106±231	-132±7/ -153±9	-96±5/ -87±3	-36±8/ -66±9
siSER-11 / siSER-11m	5719±199/ 6064±250	-75±15/ -131±14	-5761±196/ -6066±244	-117±11/ -132±13	-96±6/ -86±5	-21±5/ -46±8
siAGT-1 / siAGT-1m	5710±239/ 5868±219	-106±10/ -133±12	-5764±233/ -5910±215	-159±11/ -176±13	-86±4/ -90±3	-73±5/ -86±13
siAGT-2 / siAGT-2m	5842±149/ 6008±147	-123±9/ -133±9	-5873±147/ -6035±142	-154±8/ -161±8	-92±3/ -89±2	-62±6/ -72±5
siAGT-3 / siAGT-3m	5849±138/ 6144±201	-121±7/- 127±9	-5852±190/ -6147±194	-125±9/ -131±9	-88±6/ -84±2	-37±7/ -47±5
siAGT-4 / siAGT-4m	5661±195/ 5874±180	-102±11/ -131±8	-5712±191/ -5913±176	-154±11/ -170±10	-97±4/ -89±2	-57±5/ -81±5
Average parent / modified	5746±78/ 5939±106	-115±18/ -132±4	-5785±64/ -5982±106	-152±17/ -161±16	-92±4/ -88±2	-59±18/ -73±15

Table S5. Per-nucleotide g1-g8 energy decomposition for siRNA duplexes: Shown in units of kcal/mol are the interaction energies per base pair ΔE_i for the guide strand nucleotides g1-g8 in the seed region in parent and modified siRNA duplexes calculated based on the output from the MD simulations. Also shown are the average value of the interaction energies per base pair for the guide region positions g1-g8, $\Delta E_{1-8,av}$.

siRNA duplex	ΔE_1	ΔE_2	ΔE_3	ΔE_4	ΔE_5	ΔE_6	ΔE_7	ΔE_8	$\Delta E_{1-8,av}$
siSER-1	-5.4±1.6	-12.8±1.4	-10.8±1.4	-5.2±1.4	-10.8±1.4	-12.2±1.6	-6.6±1.8	-10.0±1.6	-9.23±3.72
siSER-1m	-5.4±3.4	-13.6±1.4	-11.0±2.4	-5.8±1.4	-11.4±1.8	-12.4±2.4	-7.4±1.4	-10.4±1.6	-9.68±3.26
siSER-2	-6.0±3.2	-11.4±1.4	-5.6±1.4	-10.0±1.4	-4.6±2.0	-5.8±1.4	-10.4±2.4	-9.2±1.6	-7.88±3.40
siSER-2m	-5.6±2.8	-12.4±2.4	-6.4±2.0	-10.4±1.6	-5.6±2.0	-6.4±1.6	-10.8±2.4	-9.4±1.6	-8.34±3.76
siSER-3	-6.0±1.6	-11.6±1.4	-10.4±1.4	-10.8±1.4	-12.2±1.4	-11.4±1.6	-6.2±1.8	-12.0±1.6	-10.08±2.94
siSER-3m	-0.2±2.0	-11.4±2.4	-10.6±2.0	-10.8±1.6	-12.4±1.4	-10.8±1.6	-6.2±2.4	-12.2±1.8	-9.33±2.74
siSER-4	-5.8±1.6	-7.0±1.4	-11.2±1.4	-5.4±1.4	-10.0±1.8	-5.4±2.4	-4.6±2.0	-5.8±1.6	-6.88±3.16
siSER-4m	-6.4±2.0	-4.0±2.0	-11.8±1.4	-6.4±2.4	-10.4±1.6	-5.8±2.4	-5.0±2.0	-6.4±2.4	-7.03±3.90
siSER-5	-6.6±1.6	-5.8±1.6	-6.4±2.0	-9.4±1.4	-6.0±1.4	-6.4±1.4	-9.6±1.4	-5.8±1.4	-7.03±3.40
siSER-5m	-6.2±2.6	-5.0±2.6	-6.6±1.8	-10.4±1.4	-6.6±2.4	-6.6±2.4	-10.2±1.4	-6.4±1.4	-7.25±3.30
siSER-6	-6.2±3.0	-10.6±2.4	-10.6±2.0	-8.8±1.4	-10.8±1.4	-12.0±1.4	-6.8±1.4	-9.4±1.4	-9.40±3.02
siSER-6m	-6.0±2.6	-10.4±2.4	-11.4±1.6	-6.8±1.4	-11.4±1.4	-12.6±2.4	-8.0±1.4	-9.8±1.4	-9.55±2.84
siSER-7	-3.6±2.2	-12.0±1.6	-8.4±2.4	-10.0±1.6	-13.6±1.6	-4.4±2.4	-11.0±1.6	-8.8±1.4	-8.98±2.14
siSER-7m	-6.6±1.8	-11.0±1.8	-5.6±1.4	-11.2±1.8	-12.8±2.4	-6.2±1.4	-10.8±1.4	-6.0±1.6	-8.90±2.66
siSER-8	-5.2±1.8	-11.4±2.4	-6.4±2.0	-11.0±2.4	-11.4±1.4	-6.8±2.4	-12.0±1.4	-10.6±1.4	-9.35±2.64
siSER-8m	-6.6±1.8	-6.2±2.4	-6.4±2.4	-6.4±2.4	-11.4±1.6	-10.2±2.4	-9.8±1.6	-7.2±1.4	-8.05±3.34
siSER-9	-5.6±1.4	-10.8±1.6	-11.2±1.4	-6.8±1.6	-12.2±1.4	-10.6±1.4	-4.8±1.4	-5.2±1.4	-8.40±4.04
siSER-9m	-5.2±1.8	-11.2±1.8	-11.8±1.6	-8.0±1.4	-12.8±1.4	-10.4±1.4	-3.2±1.6	-5.6±1.6	-9.53±2.84
siSER-10	-5.4±2.4	-5.4±1.8	-6.4±1.8	-11.2±2.4	-10.0±2.2	-5.0±2.2	-9.8±2.4	-10.4±2.4	-7.95±2.68
siSER-10m	-6.8±2.6	-6.0±1.6	-7.6±2.0	-12.6±2.0	-11.4±2.0	-5.8±1.8	-11.4±2.2	-12.2±2.2	-9.23±2.42
siSER-11	-4.0±2.0	-5.0±1.6	-6.2±1.4	-6.0±1.4	-5.2±1.6	-5.2±1.6	-5.0±1.8	-9.0±2.4	-5.70±1.48
siSER-11m	-4.4±3.2	-3.2±2.1	-7.0±1.4	-6.8±1.6	-5.8±1.6	-6.0±1.8	-6.0±1.8	-10.4±2.2	-6.20±3.44
siAGT-1	-9.6±2.6	-13.0±2.6	-10.2±2.4	-10.4±2.4	-8.0±2.4	-12.2±2.2	-5.4±1.6	-10.2±2.4	-9.88±3.08
siAGT-1m	-6.0±3.0	-9.2±2.7	-13.0±2.2	-10.6±2.0	-4.6±2.6	-11.2±2.4	-6.4±1.6	-11.0±2.0	-9.43±2.54
siAGT-2	-5.4±1.4	-11.0±1.6	-11.4±1.6	-7.0±1.6	-11.2±1.4	-4.4±2.0	-5.6±1.4	-10.4±1.4	-8.30±2.82
siAGT-2m	-5.6±1.8	-9.8±1.8	-11.4±1.4	-7.6±1.4	-11.8±2.4	-5.0±1.4	-6.2±1.6	-10.6±1.6	-8.50±2.40
siAGT-3	-5.6±2.0	-5.6±1.4	-5.6±1.4	-6.6±2.4	-6.4±2.4	-5.4±2.0	-5.2±1.4	-4.8±2.4	-5.65±0.82
siAGT-3m	-5.8±1.8	-5.8±1.6	-6.2±1.4	-7.4±1.4	-7.2±1.8	-5.8±1.4	-5.8±0.4	-5.2±1.6	-6.15±1.50
siAGT-4	-5.6±2.0	-5.6±1.4	-5.4±1.4	-11.2±2.4	-10.2±2.4	-5.8±2.0	-6.8±1.4	-10.2±2.4	-6.93±2.12
siAGT-4m	-4.4±3.0	-12.6±1.6	-6.2±2.0	-6.2±2.0	-10.0±1.8	-1.2±1.4	-6.6±1.6	-10.8±1.4	-7.25±3.70

Table S6. Per-nucleotide g1-g8 SASA decomposition for siRNA duplexes: Shown in units of Å^2 are $SASA$ values for the guide strand nucleotides g1-g8 corresponding to the seed region in parent and modified siRNA duplexes calculated based on the output from the MD simulations. Also shown are the average value of the SASA per base pair for the guide region positions g1-g8, $SASA_{1-8,av}$.

siRNA duplex	$SASA_1$	$SASA_2$	$SASA_3$	$SASA_4$	$SASA_5$	$SASA_6$	$SASA_7$	$SASA_8$	$SASA_{1-8,av}$
siSER-1	193	178	173	172	174	174	170	181	177
siSER-1m	227	174	192	186	185	164	190	195	189
siSER-2	188	186	173	183	168	177	178	174	178
siSER-2m	225	170	191	196	184	166	194	189	189
siSER-3	185	188	177	175	172	169	173	182	177
siSER-3m	245	240	189	187	183	157	187	195	198
siSER-4	193	173	186	182	186	170	153	162	176
siSER-4m	155	216	192	187	195	161	188	190	186
siSER-5	170	163	168	178	175	171	178	176	172
siSER-5m	120	201	179	190	189	160	194	191	178
siSER-6	193	180	177	168	175	171	170	178	177
siSER-6m	170	187	201	184	186	162	190	192	184
siSER-7	177	180	172	176	173	170	181	173	175
siSER-7m	127	194	171	182	185	159	198	190	176
siSER-8	181	175	178	174	183	177	174	170	177
siSER-8m	231	191	181	188	160	190	170	172	185
siSER-9	198	172	169	170	183	178	172	172	177
siSER-9m	228	165	184	185	195	165	197	191	189
siSER-10	201	167	172	181	179	170	181	180	179
siSER-10m	203	184	191	195	196	163	200	197	191
siSER-11	185	174	168	168	167	168	169	178	172
siSER-11m	154	204	192	180	184	160	191	194	182
siAGT-1	202	177	182	178	171	175	169	174	179
siAGT-1m	186	186	204	190	186	166	186	188	187
siAGT-2	200	169	166	171	180	168	177	177	176
siAGT-2m	215	163	184	185	194	158	196	191	186
siAGT-3	192	171	170	173	168	168	169	170	173
siAGT-3m	206	162	188	186	183	159	189	181	182
siAGT-4	210	183	164	168	171	173	172	177	177
siAGT-4m	231	156	181	184	180	161	192	187	184

Table S7. Sequence analysis of siRNA seed region: Number of active (+) and inactive (-) modified siRNA duplexes for bases A and U combined, and for bases G and C combined in the seed region (positions g2-g8). Fluorine modifications are highlighted in green.

Guide position	AU<br>+/-	GC<br>+/-
2	6/0 (100%/0%)	2/7 (22%/78%)
3	6/2 (75%/25%)	2/5 (29%/71%)
4	6/3 (67%/33%)	2/4 (33%/67%)
5	3/2 (60%/40%)	5/5 (50%/50%)
6	7/2 (78%/22%)	1/5 (17%/83%)
7	5/5 (50%/50%)	3/2 (60%/40%)
8	4/2 (67%/33%)	4/5 (44%/56%)

Table S8. Statistics of C2′- and C3′-endo conformation: Shown are the percentages of C2′- and C3′-endo conformations at each position on the guide strand for two extreme IC_{50} activity cases: siSER-11/siSER-11m and siSER-1/siSER-1m.

Guide position	C2′-endo/C3′-endo % (siSER-11)	C2′-endo/C3′-endo % (siSER-11m)	C2′-endo/C3′-endo % (siSER-1)	C2′-endo/C3′-endo % (siSER-1m)
1	0 / 100	7 / 93	15 / 85	89 / 11
2	0 / 100	57 / 43	0 / 100	0 / 100
3	0 / 100	0 / 100	0 / 100	0 / 100
4	0 / 100	0 / 100	0 / 100	0 / 100
5	0 / 100	0 / 100	0 / 100	0 / 100
6	0 / 100	0 / 100	0 / 100	1 / 99
7	0 / 100	0 / 100	0 / 100	0 / 100
8	0 / 100	0 / 100	0 / 100	0 / 100
9	0 / 100	0 / 100	0 / 100	0 / 100
10	0 / 100	0 / 100	0 / 100	0 / 100
11	0 / 100	0 / 100	0 / 100	0 / 100
12	0 / 100	0 / 100	0 / 100	0 / 100
13	1 / 99	34 / 66	0 / 100	0 / 100
14	0 / 100	0 / 100	0 / 100	0 / 100
15	0 / 100	0 / 100	0 / 100	0 / 100
16	0 / 100	0 / 100	0 / 100	0 / 100
17	0 / 100	0 / 100	0 / 100	0 / 100
18	0 / 100	0 / 100	0 / 100	0 / 100
19	1 / 99	3 / 97	11 / 89	51 / 49

Table S9. Position-specific interaction energy decomposition. Interaction energy decompositions for positions g2, g4, g6 in the guide strand depending on the ribose conformation (C2′-endo $\Delta E_{C2'}$ and C3′-endo $\Delta E_{C3'}$), as well as the difference in the energy between C2′-endo and C3′-endo conformations $\Delta\Delta E_{C2'-C3'}$ calculated based on the output from the MD simulations for all 15 modified siRNA sequences. The data (# of occurrences) and the percentage (%) is shown for all the nucleotides combined, and for each of them separately.

	**AUGC**	**A**	**U**	**G**	**C**
**position g2 (2′-F)**					
$\Delta E_{C2'}$, kcal/mol (# of instances: % C2′-endo)	-2.5±2.1 (1289: 18%)	-3.3±0.3 (3: 1%)	-1.1±1.0 (483: 27%)	-6.0±1.2 (142: 7%)	-2.7±1.8 (657: 29%)
$\Delta E_{C3'}$, kcal/mol (# of instances: % C3′-endo)	-4.3±2.2 (5733: 82%)	-3.1±0.7 (997: 99%)	-1.8±1.0 (1315: 73%)	-6.7±1.3 (1368: 93%)	-4.2±1.1 (1629: 71%)
$\Delta\Delta E_{C2'-C3'}$, kcal/mol		-0.2	0.7	0.7	1.5
**position g4 (2′-OMe)**					
$\Delta E_{C2'}$, kcal/mol (# of instances: % C2′-endo)	--- (0: 0%)	--- (0: 0%)	--- (0: 0%)	--- (0: 0%)	--- (0: 0%)
$\Delta E_{C3'}$, kcal/mol (# of instances: % C3′-endo)	-3.6±1.6 (7022: 100%)	-3.6±0.8 (1673: 100%)	-2.2±0.8 (2771: 100%)	-5.4±1.1 (2096: 100%)	-4.5±0.9 (482: 100%)
$\Delta\Delta E_{C2'-C3'}$, kcal/mol		---	---	---	---

Supplemental Methods

Calculation of atomic partial charges for modified siRNA duplexes: Atomic partial charges from the bsc0χOL3 (part of AMBER14SB) force field were used to model the phosphate groups and all four nucleotide bases: adenine (A), cytosine (C) guanine (G), and uracil (U). The atomic partial charges in the ribose ring due to chemical modifications (2′-F and 2′-OMe; Fig. 1 in the main part) were calculated using the RESP method [2] and the Hartree-Fock theory and the 6-31G* basis set (with Gaussian 16 package [3]). The procedure of Restrained ElectroStatic Potential (RESP) charge fitting is described in Refs. [2,4] and is implemented in the RED server (RESP ESP charge Derive Server) [5]. Several *ab initio* calculations were carried out for each fragment and for each nucleobase A, U, G, or C (6 runs per nucleobase for 2′-OMe and 2′-F; a total of 48 runs) similarly to our previous work [6]. For each atom in the ribose ring with the 2′-F and 2′-OMe modifications, we averaged their partial charges over these different partial charge calculations, so the atomic charges on the modified ribose would be the same (except for the C1′ and H1′ atoms) for any base, as is the case for the ribose ring in the canonical bsc0$_{\chi}$OL3 force field for RNA [7–10]. The calculated charges were fitted using the RESP algorithm. Instead of calculating partial charges for an entire siRNA structure fragment (i.e. phosphate plus ribose plus base), we used the ribose ring with nucleobase substituted with a formamide -NH-CHO group, as described in Ref. [4]. The formamide group resembles the immediate chemical environment for sugars linked to the bases. Atomic partial charges for the formamide-sugar system with chemical modifications (2′-F and 2′-OMe) were derived using the RED server, which automates the calculation of RESP-charges at the HF/6-31G(d) level of theory while also maintaining consistency with the bsc0χOL3 force field (see Fig. S1).

Construction of siRNA duplex structures *in silico*: The double-helical conformations of the parent siRNA duplexes were obtained using the SimRNA software package [11]. In these duplex structures, we also included the 2 nt overhangs at the 3′-end of the passenger and guide strands (Fig. 1 and Table 1 in the main text). The topology and coordinate files for each siRNA duplex were prepared using the tleap module implemented in the AMBER 20 package [12]. In each siRNA duplex, the negatively charged phosphate groups were neutralized using K^+ counterions to make the net charge of the system neutral similarly to our previous works with RNA duplexes, DNA:RNA hybrids, and single stranded RNAs and DNAs [13,14]. To construct the chemically modified siRNA duplexes, the 2′-OH groups in the ribose rings in the parent siRNA duplexes were replaced with the 2′-F and 2′-OMe modifications (Fig. 1 in the main part).

Analysis of MD simulation output: The energy and coordinate files from 1-μs all-atom MD trajectories were saved every 2 ps and extracted every 200 ps time interval for data analysis. The root mean square deviations (RMSDs) for all atoms of siRNA molecules and their end-to-end distances (X) were calculated using the VMD package [15]. For RMSD analysis, all the frames were superimposed with the initial energy-minimized structure of the siRNA duplex in question after removing all the water molecules. Snapshots of the time-averaged siRNA structures were reconstructed using the VMD package. Using these averaged structures and the Curves+ package [16] we profiled the intra-base pair (base-pairing) and inter-base pair (base-stacking) interaction parameters and the numbers of hydrogen bonds (H-bonds) per base pair. The atomic distance between heavy atoms and bond angles formed by three atoms were calculated using the PTRAJ module [17]. The Solvent Accessible Surface Area (SASA) was estimated using the LCPO algorithm [18] implemented in the CPPTRAJ module [17] in AmberTools20 [12]. The results of simulations were visualized with the VMD package [15]. The molecular mass m of siRNA duplexes was calculated using the CPPTRAJ module [17]. The molecular volume V of siRNA duplexes was estimated for the time-averaged structure

using the VolMap plugin in the VMD package [15]. The siRNA density ρ was calculated using the formula $\rho = m/V$. Pseudorotation sugar phase parameter P was calculated using Barnaba software [19] as described in Ref. [1]: $P = 180/\pi \cdot \arctan[2(\nu_4 + \nu_1 - \nu_3 - \nu_0, 3.0777\nu_2)]$, where ν_i are five torsion (pucker) angles of the furanose ring.

Hydrogen bonds, base pairing, and base stacking interactions: The ensemble average structures for each siRNA duplex (listed in Table 1 in the main text) were generated using Tcl script implemented in VMD [15]. We used the H-bond definition, according to which an H-bond is formed between a pair of donor (D) and acceptor (A) heavy atoms; if a donor–acceptor distance is less than 3.3Å and an D-H…A angle is larger than 135°. The base pairing and base stacking interactions were identified and the total numbers of base pairs and base stacks were calculated using Barnaba software [19]. The structure schematic for the calculation of the numbers of base pairs and base stackings is shown in Figs. S1C and S1D. Any two bases within the same (guide or passenger) strand were considered to be stacked if ($|\zeta_{kj}|$ and $|\zeta_{jk}|$ > 2Å) and (σ_{kj} or σ_{jk} < 2.5Å) and ($|\theta_{kj}|$ < 40°). Here, $\sigma_{ij} = \sqrt{\xi_{kj}^2 + \chi_{kj}^2}$, where the ξ- and χ-axes are in the plane of the base (ξ_{kj} and χ_{kj} are the distances between the centers of mass of the two bases along the ξ- and χ-axes, respectively) and the ζ-axis is normal to the $\xi\chi$-plane, ζ_{kj} is the distance between the centers of mass of the two bases, and θ_{kj} is the angle between the normal vectors of the two bases [19] (see Fig. S1). All the non-stacked bases were considered to be base paired if $|\theta_{kj}|$ < 60° and there exists at least one hydrogen bond (H-bond) between k-th and j-th bases (Fig. S1). *Base pairing interactions:* The base pairing interaction parameters (see Fig. S2A) comprise three translations, i.e. the shear (along the axis transverse to the base-base x-axis axis), the stretch (along the base-base y-axis axis), and the stagger (along the helical z-axis), and three rotations, i.e. the buckle (around the x-axis), the propeller (around the y-axis) and the opening (around the z-axis). *Base stacking interactions:* The base stacking interaction parameters (see Fig. S2B) involve three translations, i.e. the shift (along the x-axis), the slide (along the y-axis) and the rise (along the z-axis), and three rotations, i.e. the tilt (around the x-axis), the roll (around the y-axis) and the twist (around the z-axis).

Calculation of thermodynamic state functions: The Molecular Mechanics/Generalized Born Surface Area (MM/GBSA) approach [20] was used to calculate the internal energy, entropy, and free energy of interaction (binding energy) between the guide and passenger strands (Table S4) forming siRNA duplexes (see Table S4 and Figs. 3, 5, 6 in the main text and Fig. S5). For each dsRNA duplex, a total of 100 snapshots (100 data points) were extracted from the 1-μs MD simulation runs for the MM/GBSA-based calculations [21]. The internal energy E_{int} includes the energy of covalent bonds E_{bond}, energy of bond angles E_{ang}, energy of dihedral angles E_{dih}, electrostatic energy E_{el} and van der Waals E_{vdW}. The molecular mechanics component of energy E_{MM} is calculated by evaluating the sum, $E_{MM} = E_{int} + E_{el} + E_{vdW}$. The solvation energy E_{solv} is described in terms of the polar contribution E_{GB} and the nonpolar E_{np} contribution respectively, i.e. $E_{solv} = E_{GB} + E_{np}$. Here, E_{GB} is calculated using the Generalized Born (GB) model, and E_{np} is estimated using the solvent-accessible surface area (SASA). We set the dielectric constant of the water solvent to 80, and the dielectric constant of the solute (protein and RNA) to 1. We took E_{np} to be proportional to the constant surface area energy density equal to 0.0072 kcal·mol^{-1}·Å^{-2} [22]. Thus, the energy of a dsRNA duplex is given by $E = E_{MM} + E_{solv}$. Normal Mode Analysis (NMA) was used to estimate the entropy difference ΔS between the entropies of siRNA duplexes and the entropic

contributions from each strand [23]. For each dsRNA duplex, the binding energy ΔE and binding entropy ΔS were calculated as $\Delta E = E_d - (E_g + E_p)$ and $\Delta S = S_d - (S_g + S_p)$, where E_g, S_g, E_p, S_p, and E_d, S_d are energies and entropies of the guide strand, passenger strand and dsRNA duplex, respectively. The binding free energy ΔG at $T = 300$ K temperature was calculated using the second law of thermodynamics: $\Delta G = \Delta E - T\Delta S$. This approach was also used to profile the pairwise energy decomposition (binding energy) on per base-pair bases (see Figs 5, 6 in the main text and Fig. S5) [20,24].

Nearest Neighbor model: The Nearest-Neighbor (NN) model [25] provides a means to calculate the thermodynamic state functions, including the enthalpy change ΔE, entropy change ΔS, and free energy change ΔG, associated with the formation of the double-stranded (ds) RNA secondary structure from the two complementary RNA single-strands. For a dsRNA duplex, ΔE, ΔS and ΔG could be obtained from the following equations: $\Delta E = \Delta E_{helix} = \sum_i^N \Delta E_i + \Delta E_{init} + \Delta E_{helix\,ends}$, $\Delta S = \Delta S_{helix} = \sum_i^N \Delta S_i + \Delta S_{init} + \Delta S_{helix\,ends} + \Delta S_{symm}$, and $\Delta G = \Delta G_{helix} = \sum_i^N \Delta G_i + \Delta G_{init} + \Delta G_{helix\,ends} + \Delta G_{symm}$. In these equations, ΔE_i, ΔS_i and ΔG_i are an energy, entropy and free energy of a particular piece of the sequence, ΔE_{init}, ΔS_{init} and ΔG_{init} are the initiation correction factor for energy, entropy and free energy, respectively, $\Delta E_{helix\,end}$, $\Delta S_{helix\,end}$, $\Delta G_{helix\,end}$, are the corrections for terminating a helix with an AU or GU base pair, and ΔS_{symm} and ΔG_{symm} are the symmetry correction factors for entropy and free energy predictions, respectively (N is the total number of base pair stacks in the RNA double helix). For RNA structures with overhangs both at the 3′-end and 5′-end, the energy, entropy, and free energy changes can be calculated using the following equations: $\Delta E = \Delta E_{helix} + \Delta E_{unpair} = \sum_i^N \Delta E_i + \Delta E_{init} + \Delta E_{symm} + \sum_l^L \Delta E_{unpair,l}$, $\Delta S = \Delta S_{helix} + \Delta S_{unpair} = \sum_i^N \Delta S_i + \Delta S_{init} + \Delta S_{symm} + \sum_l^L \Delta S_{unpair,l}$, and $\Delta G = \Delta G_{helix} + \Delta G_{unpair} = \sum_i^N \Delta G_i + \Delta G_{init} + \Delta G_{symm} + \sum_l^L \Delta G_{unpair,l}$. Here, $\Delta E_{unpair,l}$, $\Delta S_{unpair,l}$ and $\Delta G_{unpair,l}$ are the energy, entropy and free energy of a particular piece of sequence with one of the bases unpaired (L is the total number of unpaired bases). Numerical values of ΔE_i, ΔS_i, ΔG_i and $\Delta E_{unpair,l}$, $\Delta S_{unpair,l}$ $\Delta G_{unpair,l}$ correspond to 1 M solution of NaCl at $T = 310.15$ K temperature [25]. As an example, for a siSER-1 (see Table 1 in the main text), the free energy change ΔG can be calculated as

$$\Delta G = 3\Delta G\begin{pmatrix}\overrightarrow{CA}\\ \overleftarrow{GU}\end{pmatrix} + 3\Delta G\begin{pmatrix}\overrightarrow{CU}\\ \overleftarrow{GA}\end{pmatrix} + 2\Delta G\begin{pmatrix}\overrightarrow{GA}\\ \overleftarrow{CU}\end{pmatrix} + 2\Delta G\begin{pmatrix}\overrightarrow{GU}\\ \overleftarrow{CA}\end{pmatrix} + \Delta G\begin{pmatrix}\overrightarrow{CG}\\ \overleftarrow{GC}\end{pmatrix} + 2\Delta G\begin{pmatrix}\overrightarrow{GC}\\ \overleftarrow{CG}\end{pmatrix} + 5\Delta G\begin{pmatrix}\overrightarrow{GG}\\ \overleftarrow{CC}\end{pmatrix} + \Delta G_{init} + \Delta G_{symm} + 2\Delta G_{helix\,end\,(AU\,end\,on\,GC\,pair)} \quad \text{(S1)}$$

In Eq. S1, the arrows indicate the 5′ to 3′ direction of the sequence. For the helix end contributions, the first base pair in the stack is the terminal base pair. By substituting the values of various quantities in Eq. (S1), we obtain $\Delta G =$ -39.5 kcal/mol. By the same token, the internal energy change ΔE is calculated as

$$\Delta E = 3\Delta E\begin{pmatrix}\overrightarrow{CA}\\ \overleftarrow{GU}\end{pmatrix} + 3\Delta E\begin{pmatrix}\overrightarrow{CU}\\ \overleftarrow{GA}\end{pmatrix} + 2\Delta E\begin{pmatrix}\overrightarrow{GA}\\ \overleftarrow{CU}\end{pmatrix} + 2\Delta E\begin{pmatrix}\overrightarrow{GU}\\ \overleftarrow{CA}\end{pmatrix} + \Delta E\begin{pmatrix}\overrightarrow{CG}\\ \overleftarrow{GC}\end{pmatrix} + 2\Delta E\begin{pmatrix}\overrightarrow{GC}\\ \overleftarrow{CG}\end{pmatrix} + 5\Delta E\begin{pmatrix}\overrightarrow{GG}\\ \overleftarrow{CC}\end{pmatrix} + \Delta E_{init} + 2\Delta E_{helix\,end\,(AU\,end\,on\,GC\,pair)} \quad (S2)$$

By substituting the values of various quantities in Eq. (S2), we obtain $\Delta E =$ -198.7 kcal/mol. The entropy change ΔS is calculated as

$$\Delta S = 3\Delta S\begin{pmatrix}\overrightarrow{CA}\\ \underleftarrow{GU}\end{pmatrix} + 3\Delta S\begin{pmatrix}\overrightarrow{CU}\\ \underleftarrow{GA}\end{pmatrix} + 2\Delta S\begin{pmatrix}\overrightarrow{GA}\\ \underleftarrow{CU}\end{pmatrix} + 2\Delta S\begin{pmatrix}\overrightarrow{GU}\\ \underleftarrow{CA}\end{pmatrix} + \Delta S\begin{pmatrix}\overrightarrow{CG}\\ \underleftarrow{GC}\end{pmatrix} + 2\Delta S\begin{pmatrix}\overrightarrow{GC}\\ \underleftarrow{CG}\end{pmatrix}$$
$$+5\Delta S\begin{pmatrix}\overrightarrow{GG}\\ \underleftarrow{CC}\end{pmatrix} + \Delta S_{init} + \Delta S_{symm} + 2\Delta S_{helix\ end\ (AU\ end\ on\ GC\ pair)} \qquad \text{(S3)}$$

By substituting the values of various quantities in Eq. (S3), we obtain $\Delta S = 0.5$ kcal/mol^{-1}K^{-1}. The NN model was used to estimate the values of ΔE, ΔS, and ΔG for siRNA duplexes.

Statistical Modeling: Parameters accessible through the all-atom MD simulations were used as input variables (model features) to characterize the relative importance of the various structural, dynamic, and thermodynamic properties of siRNA duplex at predicting their biological activity.

Training Data Set: For each duplex, feature values were generated to create a training data set, including: the free energy of the duplex, ΔG; molecular mechanics component of energy of the duplex, ΔE_{MM}; the free energy of the duplex calculated using the Generalized Born model, ΔG_{GB}; the enthalpy of the duplex calculated using the Generalized Born model, ΔE_{GB}; the nonpolar contribution of the free energy of the duplex, ΔG_{NP}; the van der Waals energy for each nucleotide, $\Delta E_{vdW,i}$, $i = 1,\ldots,21$ (index i enumerates the position for the guide strand nucleotides g1-g21); the total van der Waals energy for the seed region, $\Delta E_{vdW,1-8} = \sum_{i=1}^{8} \Delta E_{vdW,i}$; the van der Waals energy for the duplex, ΔE_{vdW}; the electrostatic energy for each nucleotide, $\Delta E_{el,i}$;the total electrostatic energy for the seed region, $\Delta E_{el,1-8} = \sum_{i=1}^{8} \Delta E_{el,i}$; the total electrostatic energy for the duplex, ΔE_{el}; the total interaction energy for each nucleotide, ΔE_i; the total interaction energy for the seed region, $\Delta E_{1-8} = \sum_{i=1}^{8} \Delta E_i$ (see Fig. 5 in the main text and Table S5); the entropic contribution to the free energy of the duplex, $T\Delta S$; the solvent accessible surface area for the whole duplex, $SASA$; the solvent accessible surface area for the guide strand in the seed region, $SASA_{1-8,av,guide}$; the solvent accessible surface area for the guide and passenger strand in the seed region, $SASA_{1-8,av,guide+passenger}$; the hydrodynamic volume of the duplex, V; the hydrodynamic volume of the guide strand in the seed region, $V_{1-8,guide}$; and the hydrodynamic volume of the guide plus passenger strand in the seed region, $V_{1-8,guide+passenger}$; the end-to-end distance for the duplex, X; the end-to-end distance for the seed region, X_{1-8}; predicted melting temperatures for each stack of three base pairs using NN parameters, $T_{m,i-(i+2)}$; predicted melting temperature for the whole duplex ($T_{m,1-19}$) and the seed ($T_{m,2-9}$) and tail regions ($T_{m,13-19}$).

Partial Least Squares Regression: Partial Least Squares Regression (PLS), also called Projection to Latent Structures, is a modeling method that overcomes limitations in standard regression methods when the number of features exceeds the number of observations or when there are strong correlations between features [26]. Like principal component regression, PLS replaces the feature matrix (**X**) with the product of an orthogonal score matrix and a loadings vector. Unlike principal component regression, where the scores matrix reflects the variance within the feature matrix, PLS builds a scoring matrix to reflect the covariance between the feature matrix and the regression vector (**Y**). PLS model fitting was done using the *plsr* R library [27]. Leave-one-out cross-validation (LOOCV) was used to identify the optimum number of components to include in the model. LOOCV measures model performance by generating a model for each observation, where that observation has been omitted from the training data, and then making a prediction for the observation using the resulting model.

Supplemental Results

Quantum chemistry calculations: Recent quantum chemistry calculation studies showed that for RNA the C3′-endo conformation is by $\Delta E \approx 1.1$ kcal/mol more favorable than the C2′-endo conformation for ribonucleotides [28]. By performing quantum chemistry calculations, we obtained similar values of the free-energy difference between the C2′-endo and C3′-endo conformations (0.7–1.4 kcal/mol). Because the characteristic temperature for transition from the lower energy C3′-endo conformation to the higher energy C2′-endo conformation $T = {}^{\Delta E}/_{k_B} \approx 352$–$705$ K is much higher than the simulation temperature (300 K), in the calculation of atomic partial changes and in the all-atom MD simulations we set all ribonucleotides (adenine A; cytosine C; guanine G; and uracil U) to be in the free-energy minimum C3′-endo conformation (see Fig. S1).

Secondary structure of parent vs. chemically modified siRNA duplexes: We generated the average structures for each siRNA duplex and analyzed them in terms of the detailed motions of intra-base pair coupling, which describes base pairing interactions, and the inter-base pair coupling, which characterizes base stacking interactions. The most "unstable" regions, which show largest structure alterations are nucleotide regions g1-g2 and g18-g19 close to the overhangs at the 3′- and 5′- end. These alterations are expected since terminal nucleotides show enhanced interstrand dissociation thereby disrupting base pairing and base stacking interactions [29,30]. Modified dsRNAs, in general, exhibit larger internal structural alterations than their parent counterparts in the nucleotide g3-g17 region (Fig. S2A, Stretch and Buckle; Fig. S2B, Roll). Some structural perturbations were also detected in the seed region around nucleotides g2-g7, albeit just for a few sequences and only in the chemically modified siRNA duplexes (Fig. S2).

Binary correlations: For the parent siRNAs, two quantity pairs, namely ln[IC_{50}] and SASA, and T_m and SASA, are strongly positively correlated, while three quantity pairs, ln[IC_{50}] and ΔE_{vdW}, ln[IC_{50}] and ΔE, T_m and ΔE, are strongly but negatively correlated (Fig. S4A). For the modified siRNAs, five quantity pairs, IC_{50} and ΔE_{solv}, ln[IC_{50}] and SASA, ln[IC_{50}] and ΔE_{solv}, T_m and SASA, and T_m and ΔE_{solv} are strongly positively correlated. By contrast, five quantity pairs, IC_{50} and ΔE_{el}, IC_{50} and ΔE, ln[IC_{50}] and ΔE_{el}, ln[IC_{50}] and ΔE, and T_m and ΔE, are strongly but negatively correlated (Fig. S4B). We obtained large negative values of Pearson correlation coefficients ($r >$ -0.81 for combined data) and low p-values ($<4.1\times10^{-5}$ for combined data) for correlations between the guide-passenger strand interaction energies (ΔE) calculated theoretically and the experimental values of duplex melting temperature (T_m) and RNAi activity *in vitro* (IC_{50}) for both parent and modified siRNAs (Fig. 3B; Fig. 3A,C in the main text, respectively). Analysis of the experimental T_m and IC_{50} data using the properties of the seed region revealed that the molecular surface of the guide strand in the seed region accessible to solvent ($SASA_{1-8,av,guide}$) is another important feature, which accounts for >80% of the variability in IC_{50} data for dsRNA duplexes (Fig. S6) and for the correlation of $SASA_{1-8,av,guide}$ with IC_{50} data (Fig. 4B in the main text).

Statistical modeling of T_m and IC_{50} data using properties of the seed region: To identify the various RNA molecular features, reflecting the structural, dynamic, and energetic properties of siRNA duplexes molecules, that determine the IC_{50} values, we carried out statistical modeling. The model trained was a PLS model, chosen due to the ability of PLS models to handle over-determined scenarios, where the

number of features outnumber the number of observations. The only parameter that needed to be tuned for PLS models is the number of components to fit. The optimum number of components was determined with LOOCV, which determined an optimum number of three components to include in the model, resulting in a cross-validation MSE of 8.28 (data not shown). The model trained on the full dataset achieved an MSE of 5.10 (Fig. S6A). To identify the features that are most predictive of IC50, the component weights were mapped back to the feature space, resulting in a linear model. The top 20 features with the largest magnitude coefficients are SASA; $T_{m,1-3}$; $T_{m,2-4}$; $T_{m,15-17}$; $SASA_{1-8,av,guide+passenger}$; $T_{m,4-6}$; $T_{m,5-7}$; $T_{m,3-5}$; $V_{1-8,guide}$; $T_{m,14-16}$; $T_{m,16-18}$; $SASA_{1-8,av,guide}$; $T_{m,6-8}$; $T_{m,17-19}$; V; $V_{1-8,guide+passenger}$; ΔE_{MM}; $T_{m,13-15}$; ΔH_{GB}; and ΔE_{el} (Fig. S6B).

The top weighted features of the model were dominated by SASA and predicted T_m's for base pair stacks in the seed region. While $SASA_{1-8,av,guide}$ showed higher correlation to changes in IC_{50} than SASA (Fig. 4 in the main text), both are highly correlated with each other and with $SASA_{1-8,av,guide+passenger}$ (all pairwise r > 0.95), which may explain why the model changed assigned different relative weights to the three when fitting to ln[IC_{50}]. The inclusion of $T_{m,1-3}$ and $T_{m,2-4}$, agrees with the observation that the energetics and sequence of position g2 influences IC_{50} (Fig. 5, Table S7). The only features in the top 10 most impactful features that are not SASA or seed T_m related are $T_{m,15-17}$ (4th largest coefficient), $V_{1-8,guide}$ (9th largest coefficient), and $T_{m,14-16}$ (10th largest coefficient). These features are in broad agreement with our MD calculated ΔE_i values for positions g15 and g17 that show the next greatest differences, after g2 and g6, between most active and less active sequences (Fig. S5). The $T_{m,14-16}$, $T_{m,15-17}$, and $T_{m,16-18}$ parameters all overlap the supplementary binding region (g13-16), which has been shown to influence miRNA affinity [31].

Supplemental References:

1. Altona, C., Geise, H.J. t, Romers, C. Conformation of non-aromatic ring Compounds—XXV: Geometry and conformation of ring D in some steroids from X-ray structure determinations. *Tetrahedron*. 1968;24(1):13-32.
2. Bayly, C.I., Cieplak, P., Cornell, W., Kollman, P.A. A well-behaved electrostatic potential based method using charge restraints for deriving atomic charges: the RESP model. *J Phys Chem*. 1993;97(40):10269-10280.
3. Frisch, M.J., Trucks, G.W., Schlegel, H.B., Scuseria, G.E., Robb, M.A., Cheeseman, J.R., Scalmani, G., Barone, V., Petersson, G.A., Nakatsuji, H., et al. Gaussian 16. Published online 2016.
4. Cieplak, P., Cornell, W.D., Bayly, C., Kollman, P.A. Application of the multimolecule and multiconformational RESP methodology to biopolymers: Charge derivation for DNA, RNA, and proteins. *J Comput Chem*. 1995;16(11):1357-1377.
5. Vanquelef, E., Simon, S., Marquant, G., Garcia, E., Klimerak, G., Delepine, J.C., Cieplak, P., Dupradeau, F.Y. RED Server: a web service for deriving RESP and ESP charges and building force field libraries for new molecules and molecular fragments. *Nucleic Acids Res*. 2011;39(suppl_2):W511-W517.
6. Maksudov, F., Kliuchnikov, E., Pierson, D., Ujwal, M.L., Marx, K.A., Chanda, A., Barsegov, V. Therapeutic phosphorodiamidate morpholino oligonucleotides: Physical properties, solution structures, and folding thermodynamics. *Mol Ther Acids*. 2023;31:631-647.
7. Zgarbová, M., Otyepka, M., Šponer, J., Mládek, A., Banáš, P., Cheatham III, T.E., Jurecka, P. Refinement of the Cornell et al. nucleic acids force field based on reference quantum chemical calculations of glycosidic torsion profiles. *J Chem Theory Comput*. 2011;7(9):2886-2902.
8. Zgarbová, M., Sponer, J., Otyepka, M., Cheatham III, T.E., Galindo-Murillo, R., Jurecka, P. Refinement of the sugar-phosphate backbone torsion beta for AMBER force fields improves the description of Z-and B-DNA. *J Chem Theory Comput*. 2015;11(12):5723-5736.
9. Pérez, A., Marchán, I., Svozil, D., Sponer, J., Cheatham III, T.E., Laughton, C.A., Orozco, M. Refinement of the AMBER force field for nucleic acids: improving the description of α/γ conformers. *Biophys J*. 2007;92(11):3817-3829.
10. Cornell, W.D., Cieplak, P., Bayly, C.I., Gould, I.R., Merz, K.M., Ferguson, D.M., Spellmeyer, D.C., Fox, T., Caldwell, J.W., Kollman, P.A. A second generation force field for the simulation of proteins, nucleic acids, and organic molecules. *J Am Chem Soc*. 1995;117(19):5179-5197.
11. Wirecki, T.K., Nithin, C., Mukherjee, S., Bujnicki, J.M., Boniecki, M. Modeling of Three-Dimensional RNA Structures Using SimRNA. In: *Protein Structure Prediction*. Springer; 2020:103-125.
12. Case, D.A., Belfon, K., Ben-Shalom, I., Brozell, S.R., Cerutti, D., Cheatham, T., Cruzeiro, V.W.D., Darden, T., Duke, R.E., Giambasu, G., et al. Amber 2020. Published online 2020.
13. Chandra, S., Arachchillage, K.G.G.P., Kliuchnikov, E., Maksudov, F., Ayoub, S., Barsegov, V., Vivancos, J.M.A. Single-molecule conductance of double-stranded RNA oligonucleotides. *Nanoscale*. 2022;14(7):2572-2577.
14. Chandra, S., Williams, A., Maksudov, F., Kliuchnikov, E., Pattiya Arachchillage, K.G.G., Piscitelli, P., Castillo, A., Marx, K.A., Barsegov, V., Artes Vivancos, J.M. Charge transport in individual short base stacked single-stranded RNA molecules. *Sci Rep*. 2023;13(1):19858.
15. Humphrey, W., Dalke, A., Schulten, K. VMD: visual molecular dynamics. *J Mol Graph*. 1996;14(1):33-38.

16. Lavery, R., Moakher, M., Maddocks, J.H., Petkeviciute, D., Zakrzewska, K. Conformational analysis of nucleic acids revisited: Curves+. *Nucleic Acids Res*. 2009;37(17):5917-5929.
17. Roe, D.R., Cheatham III, T.E. PTRAJ and CPPTRAJ: software for processing and analysis of molecular dynamics trajectory data. *J Chem Theory Comput*. 2013;9(7):3084-3095.
18. Weiser, J., Shenkin, P.S., Still, W.C. Approximate atomic surfaces from linear combinations of pairwise overlaps (LCPO). *J Comput Chem*. 1999;20(2):217-230.
19. Bottaro, S., Bussi, G., Pinamonti, G., Reißer, S., Boomsma, W., Lindorff-Larsen, K. Barnaba: software for analysis of nucleic acid structures and trajectories. *Rna*. 2019;25(2):219-231.
20. Gohlke, H., Kiel, C., Case, D.A. Insights into protein-protein binding by binding free energy calculation and free energy decomposition for the Ras-Raf and Ras-RalGDS complexes. *J Mol Biol*. 2003;330(4):891-913.
21. Hou, T., Wang, J., Li, Y., Wang, W. Assessing the performance of the MM/PBSA and MM/GBSA methods. 1. The accuracy of binding free energy calculations based on molecular dynamics simulations. *J Chem Inf Model*. 2011;51(1):69-82.
22. Harikrishna, S., Pradeepkumar, P.I. Probing the binding interactions between chemically modified siRNAs and human argonaute 2 using microsecond molecular dynamics simulations. *J Chem Inf Model*. 2017;57(4):883-896.
23. McQuarrie, D.A. *Statistical Mechanics*. Sterling Publishing Company; 2000.
24. Manoharan, M., Akinc, A., Pandey, R.K., Qin, J., Hadwiger, P., John, M., Mills, K., Charisse, K., Maier, M.A., Nechev, L., et al. Unique gene-silencing and structural properties of 2′-fluoro-modified siRNAs. *Angew Chemie*. 2011;123(10):2332-2336.
25. Zuber, J., Schroeder, S.J., Sun, H., Turner, D.H., Mathews, D.H. Nearest neighbor rules for RNA helix folding thermodynamics: improved end effects. *Nucleic Acids Res*. 2022;50(9):5251-5262.
26. Wold, S., Sjöström, M., Eriksson, L. PLS-regression: a basic tool of chemometrics. *Chemom Intell Lab Syst*. 2001;58(2):109-130.
27. Mevik, B.H., Wehrens, R., Liland, K.H. pls: Partial least squares and principal component regression. *R Packag version*. 2011;2(3).
28. Venkateswarlu, D., Lind, K.E., Mohan, V., Manoharan, M., Ferguson, D.M. Structural properties of DNA: RNA duplexes containing 2′-O-methyl and 2′-S-methyl substitutions: a molecular dynamics investigation. *Nucleic Acids Res*. 1999;27(10):2189-2195.
29. Liu, J.D., Zhao, L., Xia, T. The dynamic structural basis of differential enhancement of conformational stability by 5′-and 3′-dangling ends in RNA. *Biochemistry*. 2008;47(22):5962-5975.
30. Nikolova, E.N., Al-Hashimi, H.M. Thermodynamics of RNA melting, one base pair at a time. *RNA*. 2010;16(9):1687-1691.
31. Sheu-Gruttadauria, J., MacRae, I.J. Structural foundations of RNA silencing by Argonaute. *J Mol Biol*. 2017;429(17):2619-2639.
